# Impact of a Chokeberry (*Aronia melanocarpa* (Michx.) Elliott) Supplementation on Cardiometabolic Outcomes: A Critical Systematic Review and Meta-Analysis of Randomized Controlled Trials

**DOI:** 10.3390/nu17091488

**Published:** 2025-04-28

**Authors:** Oleg Frumuzachi, Andrei Mocan, Sascha Rohn, Laura Gavrilaș

**Affiliations:** 1 Department of Food Chemistry and Analysis, Institute of Food Technology and Food Chemistry, Technische Universität Berlin, Gustav-Meyer-Allee 25, 13355 Berlin, Germany; oleg.frumuzachi@elearn.umfcluj.ro; 2 Department of Pharmaceutical Botany, Faculty of Pharmacy, “Iuliu Hațieganu” University of Medicine and Pharmacy, 23 Gheorghe Marinescu Street, 400337 Cluj-Napoca, Romania; mocan.andrei@umfcluj.ro; 3 Department 2, Faculty of Nursing and Health Sciences, “Iuliu Hatieganu” University of Medicine and Pharmacy, 23 Gheorghe Marinescu Street, 400337 Cluj-Napoca, Romania; laura.gavrilas@umfcluj.ro

**Keywords:** chokeberry, *Aronia melanocarpa*, (poly)phenols, cardiovascular health, lipid profile, glycemic control, blood pressure, meta-analysis, randomized controlled trial

## Abstract

**Background/Objectives**: Chokeberry (*Aronia melanocarpa* (Michx.) Elliott) is a (poly)phenol-rich fruit with purported cardiometabolic benefits. However, the evidence from randomized controlled trials (RCTs) remains inconclusive. This systematic review and meta-analysis aimed to assess the effects of chokeberry supplementation on cardiometabolic outcomes, including anthropometric parameters, glycemic control, lipid profile, and blood pressure in adults. **Methods**: A systematic literature search was conducted in PubMed, Scopus, and Web of Science through January 2025. RCTs investigating chokeberry supplementation (≥2 weeks) in adults (≥18 years) with or without cardiometabolic risk factors were included. A random effects model was used to pool effect sizes, expressed as standardized mean differences (SMDs) with 95% confidence intervals (CIs). Heterogeneity was assessed using the *I*^2^ statistic, and risk of bias was evaluated with the Cochrane risk of bias 1 (RoB 1) tool. Trial sequential analysis (TSA) was performed to assess the conclusiveness of the evidence. Certainty of evidence was rated using GRADE. **Results**: Ten RCTs (*n* = 666 participants) met the inclusion criteria. Chokeberry supplementation had no significant effects on cardiometabolic outcomes under evaluation. Subgroup analysis suggested that a chokeberry supplementation could reduce total cholesterol and LDL-C in individuals with a baseline total plasma cholesterol <200 mg/dL, and systolic blood pressure with interventions, containing >50 mg/day anthocyanin, while increasing fasting blood glucose in individuals ≤50 years old. Risk of bias was unclear or high in several studies, TSA indicated inconclusive evidence for most outcomes, and the certainty of evidence was rated as very low across all cardiometabolic markers. **Conclusions**: Chokeberry supplementation did not significantly improve cardiometabolic outcomes in the general adult population. Limited evidence is given for potential lipid-lowering and blood pressure effects in specific subgroups. However, a high risk of bias accompanies these results. More robust RCTs with standardized interventions and dietary assessments are needed.

## 1. Introduction

As has already been known for years, cardiovascular diseases (CVDs) are the leading cause of morbidity and mortality worldwide, accounting for over 30% of global deaths and projected to reach 2.4 billion deaths by the year 2030 [1]. A comprehensive evidence-based analysis of 884 randomized controlled trials (RCTs) identified various (poly)phenolic compounds, including catechin, genistein, and quercetin, as beneficial micronutrients for cardiometabolic health [2]. While both observational and interventional studies have consistently associated a higher (poly)phenol intake with improved lipid metabolism, glycemic control, and blood pressure regulation, highlighting their potential as natural therapeutic agents for cardiovascular and metabolic diseases [3,4], several limitations affect effectiveness of (poly)phenols. Unlike pharmaceutical drugs, (poly)phenolic supplements are broad-spectrum compounds with moderate or weak activity, lacking the high specificity required for a targeted therapeutic action [5]. Additionally, their low bioavailability, rapid metabolism, and quick elimination require high or very regularly given doses to achieve significant effects, which may not always be practical or safe [6]. Furthermore, the nutritional composition of natural foods is influenced by ecophysiological factors such as climate, soil, harvest date, post-harvesting handling, and storage conditions [7,8]. Accordingly, extracts made from such raw plant materials, as primarily used in (or as) supplements, suffer from the same variability in concentration and efficacy, posing challenges for standardization.

Berries, such as blueberries, blackberries, and blackcurrants, are among the richest sources of (poly)phenols, particularly anthocyanins [9]. A systematic review and meta-analysis found that a higher anthocyanin intake was significantly linked to reduced CVD risk, improved high-density lipoprotein cholesterol (HDL-C) levels, and lower concentrations of markers of oxidative stress and inflammation [10,11]. Among dark-colored berries, black chokeberry (*Aronia melanocarpa* (Michx.) Elliott) represents an interesting functional food due to its high (poly)phenol content [12]. It is a deciduous shrub from the Rosaceae family, typically reaching heights of 0.5–1 m. It is native to eastern North America but is now widely cultivated across Europe. Its fruit, small purplish-black berries (technically pomes), matures in mid-to-late summer and tends to shrivel post-ripening despite its juicy nature [13]. Nutritionally, chokeberries are characterized by a low fat and protein content but a high concentration of dietary fiber, particularly in the pomace, which constitutes up to 70% of its dry matter [14]. They also contain simple carbohydrates (fructose, glucose), essential minerals (e.g., potassium, calcium, phosphorus), trace elements (zinc, iron, selenium), and various vitamins, including B, C, K, tocopherols, and carotenoids. These berries are particularly rich in anthocyanins and other (poly)phenols, giving them their intense color and bioactivity. Their total (poly)phenolic compound content can reach up to 37,600 mg/kg dry weight [15,16]. The anthocyanin profile is dominated by cyanidin glycosides, such as cyanidin-3-*O*-galactoside and cyanidin-3-*O*-arabinoside, while their flavonol content includes derivatives of quercetin and, to a lesser extent, myricetin and kaempferol. The fruit also contains notable levels of procyanidins, chlorogenic and neochlorogenic acids, and other phenolic acids like caffeic, ferulic, and protocatechuic acid [17].

Different in vitro and in vivo animal studies have suggested that chokeberry supplementation improves blood pressure [18], reduces low-density lipoprotein cholesterol (LDL-C) [19], enhances insulin sensitivity [20], and exerts strong antioxidant and anti-inflammatory effects [21], making it a promising natural intervention for cardiometabolic health and disease risk reduction. Nonetheless, chokeberry (poly)phenols, particularly anthocyanins and proanthocyanidins, have poor absorption in the gastrointestinal tract, and a significant portion of these compounds passes unaltered into the large intestine [22,23,24,25]. Even when being absorbed, anthocyanins and flavonols undergo a fast and extensive metabolism through glucuronidation, sulfation, and methylation, which reduces their biological activity [26]. Lastly, the (poly)phenol profile of chokeberry products can degrade during processing, storage, and digestion. Anthocyanins, for example, are sensitive to pH, light, and temperature, reducing their effectiveness in processed forms like juices or supplements [27].

Despite the existing limitations, human clinical trials on chokeberry supplementation have suggested potential cardiometabolic health benefits, particularly in improving blood pressure and lipid profiles. However, the findings are inconsistent [28,29,30]. Several meta-analyses tried to systematically evaluate the available evidence, indicating that chokeberry supplementation may indeed contribute to lowering some of the cardiometabolic outcomes [31,32,33]. Likewise, the conclusions from these meta-analyses were not entirely aligned, reflecting differences in the selection of studies, statistical approaches, and overall methodological quality of meta-analyses. These discrepancies indicate the need for a more comprehensive and methodologically rigorous meta-analysis to provide a clearer and potentially more conclusive evaluation of chokeberry supplementation effects on cardiometabolic health.

Consequently, the aim of the present systematic review and meta-analysis was to critically evaluate whether chokeberry supplementation improves key cardiometabolic outcomes, including anthropometric parameters (body weight [BW], body mass index [BMI], waist circumference [WC]), glycemic parameters (fasting blood glucose [FBG], insulinemia, glycated hemoglobin [HbA1c], homeostatic model assessment for insulin resistance [HOMA-IR]), lipid profile (triacylglycerols [TAGs], total cholesterol [TC], LDL-C, HDL-C), and blood pressure (systolic [SBP] and diastolic [DBP]) in healthy or unhealthy adults (≥18 years old) as assessed through parallel or crossover RCTs with a duration of ≥2 weeks. Additionally, this study aimed to assess the methodological quality of included trials using the Cochrane risk of bias 1 (RoB 1) tool, evaluate the conclusiveness of cumulative evidence through trial sequential analysis (TSA), and determine the overall certainty of evidence using the grades of recommendation, assessment, development, and evaluation (GRADE) approach.

## 2. Materials and Methods

### 2.1. The Literature Search

The protocol for this systematic review and meta-analysis was prospectively preregistered in PROSPERO (registration number: CRD42025636417). This study adhered to the Preferred Reporting Items for Systematic Reviews and Meta-Analyses (PRISMA) 2020 guidelines [34]. A systematic search of the published literature was performed across multiple online databases, including PubMed, Scopus, and Web of Science, from their inception until 10 January 2025, without any restrictions, using relevant keywords (Supplementary Table S1). The screening process was carried out independently by two investigators, with any disagreements resolved through discussion. Additionally, literature screening using the backward and forward snowballing approach was implemented in order to conduct searches more efficiently and reliably.

### 2.2. Inclusion and Exclusion Criteria

Following the PICOS framework (population, intervention, comparison, outcomes study design), eligible studies included randomized controlled trials (RCTs) conducted on adults (≥18 years) that examined the effects of chokeberry supplementation in various forms—such as whole berries (fresh, dried, or freeze-dried), juice, chokeberry-based supplements (capsules, powders, or extracts), and food or beverages enriched with chokeberry derivatives—compared with a placebo group. The outcomes assessed included cardiometabolic health markers, such as anthropometric parameters (BW, BMI, WC), lipid profile (TAG, TC, LDL-C, HDL-C), glycemic parameters (FBG, insulinemia, HbA1c, HOMA-IR), and blood pressure (SBP and DBP). Eligible trials should have followed a parallel or crossover randomized-controlled design and had a minimum duration of two weeks, focusing on either healthy adults or those at risk for cardiometabolic conditions.

Studies were excluded when they involved individuals under 18 years of age, pregnant or lactating women, lacked a control group or unmatched placebo, did not report cardiometabolic health-related outcomes, or were non-randomized, observational, animal, or in vitro studies. Reviews, protocol articles, conference abstracts, and case reports were also excluded, along with trials lasting less than two weeks. Additionally, studies that did not report mean differences of change and standard deviations (SDs) for outcomes or provided insufficient data to calculate these values were not considered.

### 2.3. Data Extraction

Data from the selected full-text articles were extracted by one investigator and independently verified by a second investigator. The extracted information included the first author’s name, publication year, country where the trial was conducted, participant characteristics (age, sex, health status, BMI, and sample size), details of the intervention (type, dose, and duration), the comparator (type of placebo), (poly)phenol intake data, and pre- and post-intervention values for selected outcomes (mean difference of change and SD or other data that could help in calculating mean difference of change and SD). In studies where two intervention groups (e.g., intervention A with dose 1 and intervention B with dose 2) shared a common control group, the control group data were treated as a single entity for both intervention arms, aligning with the study’s design. Each effect size calculation was conducted separately to compare each intervention arm against the shared control group.

### 2.4. Assessment of Risk of Bias and Certainty of Evidence

The risk of bias in the included studies was independently evaluated by two investigators using the RoB 1 tool [35]. This assessment considered key methodological domains, including random sequence generation, carry-over effects in crossover trials, allocation concealment, blinding of participants and personnel, blinding of outcome assessment, completeness of outcome data, and selective reporting. Each domain was classified as having a “low risk of bias”, “unclear risk”, or “high risk of bias”, and an overall risk of bias was determined for each study. Any disagreements between the investigators were resolved through discussion and consensus.

To assess the certainty of the evidence, two investigators independently evaluated each meta-finding using the GRADE approach [36]. By default, RCTs were initially assigned a high certainty of evidence, which could then be downgraded based on predefined criteria, including risk of bias, inconsistency, indirectness, imprecision, and other factors such as publication bias. The final certainty of evidence was categorized as high, moderate, low, or very low. As with the risk of bias assessment, any discrepancies in the GRADE evaluation were resolved by consensus between the investigators.

### 2.5. Statistical Analysis

Mean difference of change and SDs of outcome were extracted from both the intervention and the control groups. When mean values and SDs of changes were not reported, they were calculated using baseline (*M_b_*, *SD_b_*) and post-intervention (*M_f_*, *SD_f_*) values, applying the following formulae: $Mean=M_{b}-M_{f}$ and $SD=\surd ({SD}_{b}^{2}+{SD}_{f}^{2})-(2\times R\times {SD}_{b}\times {SD}_{f})$. To confirm the robustness of the results, all analyses were repeated using correlation coefficients of 0.2 and 0.8, ensuring that findings were not influenced by the initially chosen coefficient (R = 0.5) [37]. In cases where standard error of the mean (SEM) was reported instead of SD, the SD was derived using the formula SD=SEM×√n, where *n* represents the sample size of each group [38]. The meta-analysis reported findings using the standardized mean difference (SMD) and its 95% confidence interval (CI) as the effect size. The level of statistical heterogeneity among studies was assessed using the *I*^2^ statistic [39]. Predefined subgroup analyses were conducted to compare intervention efficacy based on participants’ health status (healthy [defined as TC < 200 mg/dL, SBP < 120 mmHg, and DBP ≤ 89 mmHg] vs. cardiometabolic disease [defined as TC ≥ 200 mg/dL, SBP ≥ 120 mmHg, and/or DBP > 89 mmHg]), intervention type (chokeberry extract and/or powder vs. juice), anthocyanin dosage (≤50 mg/day vs. >50 mg/day), trial duration (≤8 weeks vs. >8 weeks), and participant age (≤50 years vs. >50 years). Sensitivity analyses were also performed to evaluate the influence of individual trials on the overall effect size. All statistical analyses were conducted using RevMan version 5.4 and R Studio version 2024.04.2 + 764 (RStudio PBC, Boston, MA, USA).

Nonetheless, TSA was conducted using TSA software (version 0.9.5.10 Beta, The Copenhagen Trial Unit, Centre for Clinical Intervention Research, The Capital Region, Copenhagen University Hospital, Rigshospitalet, 2021) to assess the robustness of the cumulative evidence and determine whether the available data were sufficient to draw definitive conclusions [40]. Depending on the outcome, conventional boundaries and alpha-spending boundaries were applied to control for type I and type II errors. The fixed significance level (α) was set at 5%, and the power was set at 80% to ensure adequate sensitivity in detecting a true effect. The O’Brien–Fleming alpha-spending function was used to adjust for repeated significance testing over accumulating data, preventing inflated type I error rates. The required information size (RIS) was estimated based on the diversity-adjusted model variance.

## 3. Results

### 3.1. Literature Search and Characteristics of Included Studies

**Figure 1:**
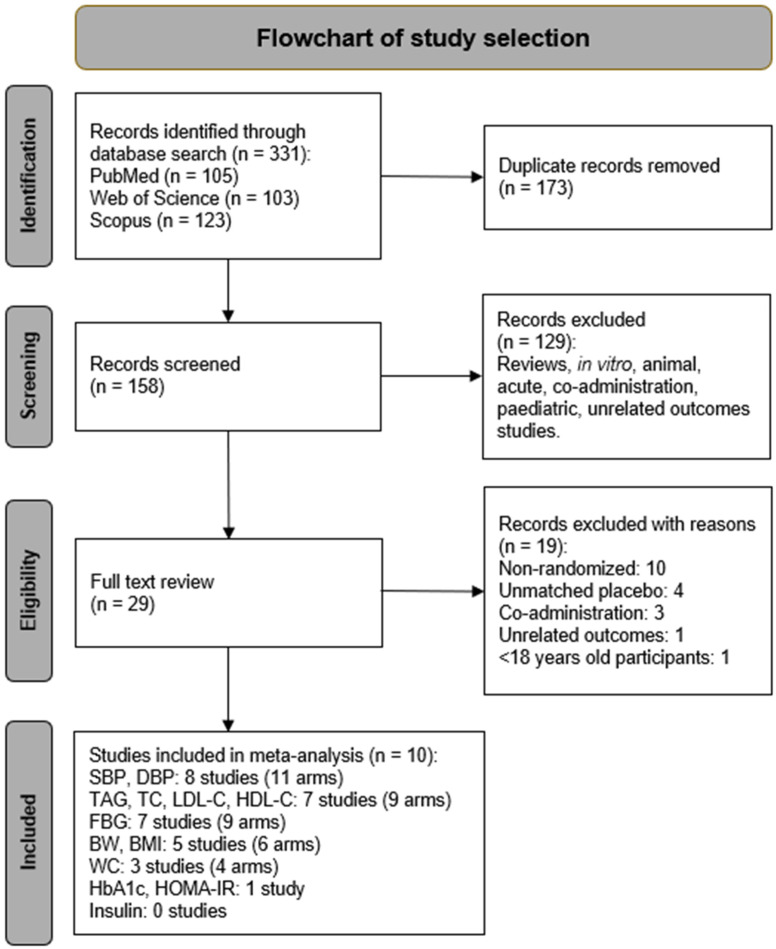
The PRISMA flowchart outlining the process of selecting studies for inclusion in systematic review and meta-analysis.

The study selection process is depicted in Figure 1. A total of 331 studies were identified through a systematic search of PubMed, Scopus, and Web of Science databases. Following the removal of 173 duplicate records, the remaining studies underwent title and abstract screening, during which 129 irrelevant studies were excluded. This left 29 publications for full-text assessment, of which 19 studies were excluded based on the following criteria: ten trials were non-randomized intervention trials [28,29,30,41,42,43,44,45,46,47], four trials had unmatched placebo [48,49,50,51], three trials used co-administration [52,53,54], and one trial each focused on unrelated outcomes [55] and participants under 18 years of age [56]. Ultimately, ten trials met the inclusion criteria and were incorporated into this systematic review and meta-analysis. The characteristics of all included RCTs are summarized in Table 1.

Briefly, nine studies were randomized [57,58,59,60,61,62,63,64,65], while one study was semi-randomized [66]; eight studies were parallel trials [57,58,59,60,61,63,64,66], while two studies were crossover trial [62,65]; finally, eight studies were double-blinded [57,58,59,61,63,64,65,66], while two studies were single-blinded [60,62]. The included studies were published between 2007 [66] and 2024 [58,60], with trials conducted in Denmark [65], Finland [62], the Netherlands [57,60], New Zealand [59], Poland [66], Serbia [63], the United Kingdom [61], and the USA [58,64]. Most studies included mixed participants, except for two studies that included only males [59,65], and another one that included only females [60]. The participants’ health status varied, with five studies focusing on healthy individuals [57,58,59,60,62,64], two studies on individuals with CVD [63,66], and three on those with pre- or mild hypertension/hypercholesterolemia [61,62,65]. The mean age of participants ranged from 24 [59] to 66 [66]. The studies covered a diverse range of BMI values, from a mean of 21.21 kg/m^2^ [60] to 29.40 kg/m^2^ [57], indicating a modest variation in body composition among study populations.

**Table 1:** Characteristics of included randomized controlled trials (RCTs) evaluating chokeberry supplementation impact on cardiometabolic outcomes. Abbreviations: cardiovascular disease (CVD), body weight (BW), body mass index (BMI), waist circumference (WC), triacylglycerol (TAG), total cholesterol (TC), low-density lipoprotein cholesterol (LDL-C), high-density lipoprotein cholesterol (HDL-C), fasting blood glucose (FBG), systolic blood pressure (SBP), diastolic blood pressure (DBP), gallic acid equivalents (GAE), cyanidin-3-glucoside equivalents (C3GE), weeks (wk), d (days), no data (ND).

Study (Year), Country	Study Design	Participants Characteristics	(Poly)phenols Intake		Investigational Approach				Duration	Measured Outcomes
			Baseline	End of trial	Type	(*n*)	Intervention	Control		
Naruszewicz et al. (2007), Poland [66]	Randomized, double-blinded, placebo-controlled, parallel trial	CVD subjects Age: 65.97 ± 8.01 years BMI: 26.70 ± 3.01 kg/m^2^ Sex: mixed (11 F/33 M)	ND	ND	Extract, capsules	Intervention: 22 Control: 22	255 mg/d (127.5 proanthocyanidins, 63.75 mg anthocyanins, 22.95 mg phenolic acids)	Placebo (maltodextrin)	6 wk	BMI, TAG, TC, LDL-C, HDL-C, FBG, SBP, DBP
Loo et al. (2016), Finland [62]	Randomized, single-blinded, placebo-controlled, crossover trial	Pre- or mild hypertensive subjects Age: 55.80 (41–69) years BMI: 25.90 ± 3.30 kg/m^2^ Sex: mixed (23 F/14 M)	Intervention and control (mg): - Phenolic acids = 630 ± 327; - Quercetin = 3.6 ± 2.5; - Total (poly)phenols = 633.6.	Intervention (mg): - Phenolic acids = 597 ± 281; Quercetin = 3.6 ± 2.8; - Total (poly)phenols = 600.6. Control (mg): - Phenolic acids = 586 ± 297; Quercetin = 3.9 ± 4.0; - Total (poly)phenols = 589.9.	Juice and chokeberry powder	Intervention: 37 Control: 37	300 mL/d (966 mg anthocyanins, 576 mg proanthocyanidins, 351 mg phenolic acids, and 55 mg flavonoids) and 3 g/d powder (169 mg proanthocyanidins, 58 mg anthocyanins, 16 mg phenolic acids, and 3.3 mg flavonoids)	Placebo juice (water, sugar syrup [12 g], and table sugar [7 g]) and placebo powder (wheat flour [0.8 g], rice powder [1.7 g], and cane sugar [0.5 g])	8 wk	BW, BMI, TAG, TC, LDL-C, HDL-C, FBG, SBP, DBP
Xie et al. (2017), USA [64]	Randomized, double-blinded, placebo-controlled, parallel trial	Healthy participants, former smokers Age: 34.95 ± 3.68 years BMI: 26.29 ± 1.27 kg/m^2^ Sex: mixed (25 F/24 M)	Intervention (mg/d): - Isoflavones = 8.5 ± 2.6; - Flavanols = 81.7 ± 18.7; - Flavones = 50.3 ± 27.2; - Flavanones = 22.4 ± 10.7; - Flavan-3-ols = 26.0 ± 6.9; - Anthocyanins = 26.6 ± 4.8; - Proanthocyanins = 418 ± 150; - Total (poly)phenols = 633.5. Control (mg/d): - Isoflavones = 3.9 ± 2.7; - Flavanols = 73.9 ± 20.0; - Flavones = 54.1 ± 29.1; - Flavanones = 28.8 ± 11.4; - Flavan-3-ols = 20.6 ± 7.4; - Anthocyanins = 18.2 ± 5.2; - Proanthocyanins = 222 ± 162; - Total (poly)phenols = 421.5.		Extract, capsules	Intervention: 25 Control: 24	500 mg/d (45.1 mg anthocyanins, 41.9 mg proanthocyanidins, and 35.7 mg hydroxycinnamic acids)	Placebo (rice powder with 0.2% beet juice concentrate)	12 wk	BW, BMI, WC, TAG, TC, LDL-C, HDL-C, SBP, DBP
Istas et al. (2019), New Zealand [59]	Randomized, double-blinded, placebo-controlled, parallel trial	Healthy subjects Age: 24 ± 5.30 years BMI: 23 ± 2.10 kg/m^2^ Sex: males (66)	Arm 1 (mg): - Total (poly)phenols = 606 ± 76; - Anthocyanins = 34 ± 8.7; - Flavan-3-ols = 130 ± 20; - Proanthocyanidins = 78 ± 16; - Flavonols = 42 ± 6.1; - Flavones = 3 ± 0.6; - Phenolic acids = 209 ± 45; - Stilbenes = 0.1 ± 0.02; - Other = 102 ± 6.2. Arm 2 (mg): - Total (poly)phenols = 568 ± 92; - Anthocyanins = 20 ± 5.5; - Flavan-3-ols = 153 ± 40; - Proanthocyanidins = 52 ± 10; - Flavonols = 45 ± 9; - Flavones = 3.2 ± 0.7; - Phenolic acids = 180 ± 36; - Stilbenes = 0.2 ± 0.1; - Other = 115 ± 11. Control (mg): - Total (poly)phenols = 419 ± 52; - Anthocyanins = 11 ± 4; - Flavan-3-ols = 71 ± 17; - Proanthocyanidins = 46 ± 12; - Flavonols = 37 ± 4.2; - Flavones = 3.8 ± 0.8; - Phenolic acids = 157 ± 36; - Stilbenes = 0.1 ± 0.05; - Other = 93 ± 5.9.	Arm 1 (mg): - Total (poly)phenols = 465 ± 73; - Anthocyanins = 31 ± 9.3; - Flavan-3-ols = 74 ± 13; - Proanthocyanidins = 87 ± 24; - Flavonols = 36 ± 0.7; - Flavones = 3.6 ± 35; - Phenolic acids = 149 ± 6.7; - Stilbenes = 0.1 ± 0.03; - Other = 84 ± 8.7. Arm 2 (mg): - Total (poly)phenols = 584 ± 84; - Anthocyanins = 24 ± 7.3; - Flavan-3-ols = 163 ± 37; - Proanthocyanidins = 70 ± 13; - Flavonols = 44 ± 0.7; - Flavones = 3.4 ± 34; - Phenolic acids = 156 ± 5.2; - Stilbenes = 0.3 ± 0.2; - Other = 123 ± 14. Control (mg): - Total (poly)phenols = 553 ± 86; - Anthocyanins = 19 ± 7; - Flavan-3-ols = 139 ± 44; -Proanthocyanidins = 56 ± 11; - Flavonols = 39 ± 1; - Flavones = 5 ± 42; - Phenolic acids = 192 ± 5; - Stilbenes = 0.2 ± 0; - Other = 103 ± 4.	Extract and powder, capsules	Arm 1: 23 Arm 2: 23 Control: 20	Arm 1: 500 mg/d extract (71 mg phenols, 30 mg anthocyanins, 16 mg proanthocyanidins) Arm 2: 500 mg/d powder (4.8 mg phenols, 3.6 mg anthocyanins, 3.3 mg proanthocyanidins)	Placebo (maltodextrin)	12 wk	BW, TAG, TC, HDL-C, LDL-C, FBG, SBP, DBP
Pokimica et al. (2019), Serbia [63]	Randomized, double-blinded, placebo-controlled, parallel trial	High CVD risk subjects Age: 40.6 ± 7.1 years BMI: 27.43 (2.81) kg/m^2^ Sex: mixed (52 F/32 M)	ND	ND	Juice	Arm 1: 27 Arm 2: 28 Control: 29	Arm 1: 100 mL/d (1177.11 GAE, 113.3 C3GE) Arm 2: 100 mL/d (294.28 GAE, 28.3 mg C3GE)	Placebo (similar appearance [color, flavor], the same nutritional composition [sugars, minerals, vitamins, organic acids], without (poly)phenols)	4 wk	BMI, WC, TAG, TC, LDL-C, HDL-C, FBG, SBP, DBP
Ahles et al. (2020), The Netherlands [57]	Randomized, double-blinded, placebo-controlled, parallel trial	Healthy subjects Age: 53 ± 5.75 years BMI: 29.40 ± 2.69 kg/m^2^ Sex: mixed (65 F/36 M)	ND	ND	Extract, capsules	Arm 1: 35 Arm 2: 34 Control: 32	Arm 1: 150 mg/d (27 mg anthocyanins) Arm 2: 90 mg/d (16 mg anthocyanins)	Placebo (maltodextrin)	24 wk	SBP, DBP
Le Sayec et al. (2022), United Kingdom [61]	Randomized, double-blinded, placebo-controlled, parallel trial	Healthy prehypertensive subjects Age: 56.20 ± 8.81 years BMI: 24.70 ± 3.16 kg/m^2^ Sex: mixed (55 F/47 M)	Intervention (mg): - Anthocyanins = 77 ± 62.1; - Flavan-3-ols = 272 ± 367; - Proanthocyanins = 211 ± 153; - Flavanones = 20.4 ± 27.7; - Flavones = 4.5 ± 4.5; - Flavonols = 74 ± 53.2; - Isoflavonoids = 5.5 ± 13.4; - Lignans = 16.9 ± 38.7; - Stilbenes = 0.4 ± 0.6; - Phenolic acids = 583 ± 365; - Other (poly)phenols = 99.1 ± 266; - Total (poly)phenols = 1364 ± 705. Control (mg): - Anthocyanins = 59.2 ± 68.9; - Flavan-3-ols = 334 ± 281; - Proanthocyanins = 308 ± 424; - Flavanones = 20.9 ± 24.2; Flavones = 5 ± 7.5; - Flavonols = 85.1 ± 73.4; - Isoflavonoids = 1.9 ± 4; - Lignans = 9.6 ± 22.5; - Stilbenes = 0.3 ± 0.4; - Phenolic acids = 691 ± 585; - Other (poly)phenols = 222 ± 916; - Total (poly)phenols = 1738 ± 1287.	Intervention (mg): - Anthocyanins = 61.2 ± 56.9; - Flavan-3-ols = 232 ± 233; - Proanthocyanins = 241 ± 184; - Flavanones = 17.8 ± 30; - Flavones = 6.8 ± 7.3; - Flavonols = 56.7 ± 34.5; - Isoflavonoids = 5.8 ± 11.3; - Lignans = 9.4 ± 16.8; - Stilbenes = 0.5 ± 0.6; - Phenolic acids = 581 ± 547; - Other (poly)phenols = 46.2 ± 84.1; - Total (poly)phenols = 1258 ± 652. Control (mg): - Anthocyanins = 73.3 ± 85.7; - Flavan-3-ols = 322 ± 283; - Proanthocyanins = 320 ± 281; - Flavanones = 17 ± 25.6; -Flavones = 5.8 ± 11.6; - Flavonols = 72.8 ± 41.9; - Isoflavonoids = 3.7 ± 9.3; - Lignans = 21.3 ± 51.5; - Stilbenes = 0.3 ± 0.6; - Phenolic acids = 695 ± 706; - Other (poly)phenols = 55.4 ± 70.7; - Total (poly)phenols = 1586 ± 998.	Extract, capsules	Intervention: 51 Control: 51	500 mg/d (31.8 mg proanthocyanidins, 23.25 anthocyanins)	Placebo (maltodextrin)	12 wk	BW, BMI, TAG, TC, LDL-C, HDL-C, FBG, SBP, DBP
Sangild et al. (2023), Denmark [65]	Randomized, double-blinded, placebo-controlled, crossover trial	Healthy pre-hypercholesterolemia subjects Age: 49.8 ± 9.7 years BMI: 27.2 ± 3.3 kg/m^2^	ND	ND	Powder, capsules	Intervention: 51 Control: 44	5 g/d (150 mg anthocyanins)	Placebo (microcrystalline cellulose)	12 wk	FBG, HbA1c, HOMA-IR, SBP, DBP
Chamberlin et al. (2024), USA [58]	Randomized, double-blinded, placebo-controlled, parallel trial	Healthy subjects Age: 33.70 ± 7.25 years BMI: 25.95 ± 5.03 kg/m^2^ Sex: mixed (8 F/6 M)	ND	ND	Juice	Intervention: 7 Control: 7	100 mL/d (623.1 mg phenolic acids, 85.02 mg anthocyanins, 8.84 mg flavonols)	Placebo (matched in taste and color, low in (poly)phenols)	4 wk	TAG, TC, LDL-C, HDL-C, FBG
Lackner et al. (2024), The Netherlands [60]	Randomized, single-blinded, placebo-controlled, parallel trial	Healthy subjects Age: 25.75 ± 4.85 years BMI: 21.21 ± 2.15 kg/m^2^ Sex: females (37)	Intervention (mg): total (poly)phenols = 663.70 ± 502.06 Control (mg): total (poly)phenols = 771.60 ± 646.22	Intervention (mg): total (poly)phenols = 557.40 ± 490.28 Control (mg): total (poly)phenols = 899.10 ± 1126.71	Juice	Intervention: 19 Control: 18	200 mL/d (1666 mg (poly)phenols)	Placebo (similar in taste, color, smell, and texture, (poly)phenol-free)	6 wk	BW, WC

Among the studies that reported participants’ (poly)phenol intake, the baseline levels of intake varied across individuals. The baseline reported total (poly)phenol intake in the control groups varied from 419 mg [59] to 1738 mg [61], while the baseline reported total (poly)phenol intake in the intervention groups varied from 568 mg [59] to 1364 mg [61]. Throughout the trials, participants in the intervention groups consumed chokeberry in different forms, including extracts and powders (in capsules), or juices: six studies used chokeberry extracts and/or powders [57,59,61,64,65,66], while four studies utilized chokeberry juices [58,60,62,63]. The supplementation dosage varied considerably, with the lowest dose being 90 mg/day (capsules) [57] and the highest at 300 mL/day (juice) [62]. The quantity of anthocyanins provided in the interventions ranged from as low as 3.6 mg/day (powder) [59] to 966 mg/day (juice) [62]. Additionally, all trials instructed participants to avoid foods rich in (poly)phenols, such as blueberries, blackcurrants, elderberries, and cranberries. At the end of the trials, (poly)phenol intake levels also showed differences between intervention and control groups. The total end-of-trial (poly)phenol intake in the control groups varied from 553 mg [59] to 1586 mg [61], while the total end-of-trial (poly)phenol intake in the intervention groups varied 465 mg [59] to 1258 mg [61]. However, five trials did not report the (poly)phenol intake among the participants [57,58,63,65,66], and one study provided some values without specifying the period to which they corresponded [64].

The duration of trials ranged from a minimum of 4 weeks [58,63] to a maximum of 24 weeks [57]. The evaluated outcomes across selected studies included WC (three studies, four arms [60,63,64]), BW and BMI (five studies, six arms [61,62,63,64,66]), FBG (seven studies, nine arms [58,59,61,62,63,65,66]), TAG, TC, LDL-C, and HDL-C (seven studies, nine arms [58,59,61,62,63,64,66]), and SBP and DBP (eight studies, eleven arms [57,59,61,62,63,64,65,66]) (*n* = 666). Most studies assessed multiple outcomes simultaneously, providing comprehensive information about the effects of chokeberry supplementation on cardiometabolic health markers. However, as only one study investigated the effects of chokeberry supplementation on HbA1c and HOMA-IR [65], and none on insulin levels, a meta-analysis was not performed for these outcomes.

### 3.2. Impact of Chokeberry on Anthropometric Parameters

**Figure 2:**
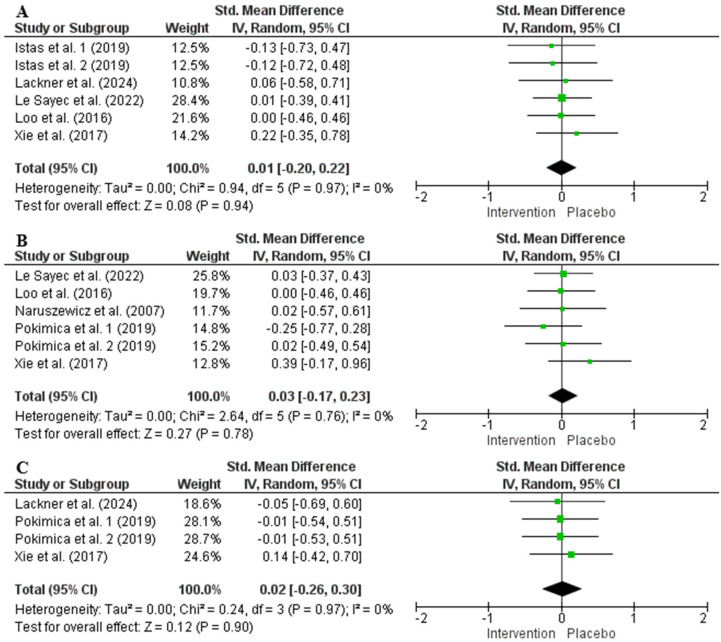
Forest plot representation of RCTs evaluating the impact of chokeberry supplementation on anthropometric parameters [59,60,61,62,63,64,66] ((**A**): body weight, (**B**): BMI, (**C**): waist circumference).

The random effects model analysis demonstrated that chokeberry interventions had no significant effect on BW (176 participants in the intervention group and 167 participants in the control group, SMD = 0.01, 95% CI: −0.20 to 0.22, *p* = 0.94, *I*^2^ = 0%), BMI (188 participants in intervention and 189 participants in control groups, SMD = 0.03, 95% CI: −0.17 to 0.23, *p* = 0.78, *I*^2^ = 0%), or WC (99 participants in intervention and 100 participants in control groups, SMD = 0.02, 95% CI: −0.26 to 0.30, *p* = 0.90, *I*^2^ = 0%) (Figure 2). Sensitivity analysis indicated that removing any single study did not substantially alter the overall effect size for anthropometric parameters. Additionally, subgroup analysis based on participants’ health status, intervention type, anthocyanin dosage, trial duration, and age did not show significant differences in BW and BMI (Supplementary Table S2). Due to the limited number of included studies, subgroup analysis for WC was not conducted.

### 3.3. Impact of Chokeberry on FBG

**Figure 3:**
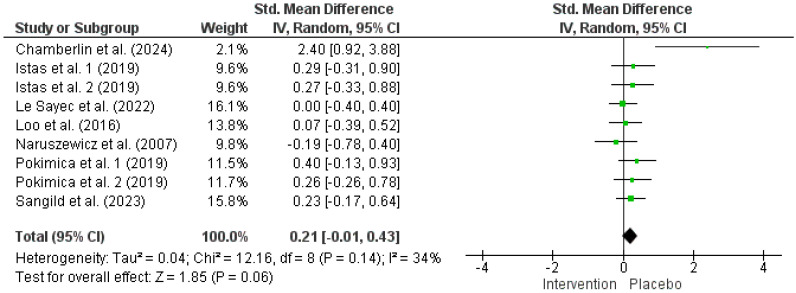
Forest plot representation of RCTs evaluating the impact of chokeberry supplementation on fasting blood glucose [58,59,61,62,63,65,66].

The random effects model analysis revealed that chokeberry interventions did not have a significant impact on FBG (267 participants in the intervention group and 256 in the control group, SMD = 0.21, 95% CI: −0.01 to 0.43, *p* = 0.06, *I*^2^ = 34%) (Figure 3). Sensitivity analysis confirmed that excluding any single study did not notably influence the overall effect size. Subgroup analysis indicated that, in participants under 50 years old, chokeberry interventions significantly increased FBG compared with the placebo (Supplementary Table S2). However, no other significant differences were observed based on participants’ health status, intervention type, anthocyanin dosage, or trial duration.

### 3.4. Impact of Chokeberry on Lipid Profile

**Figure 4:**
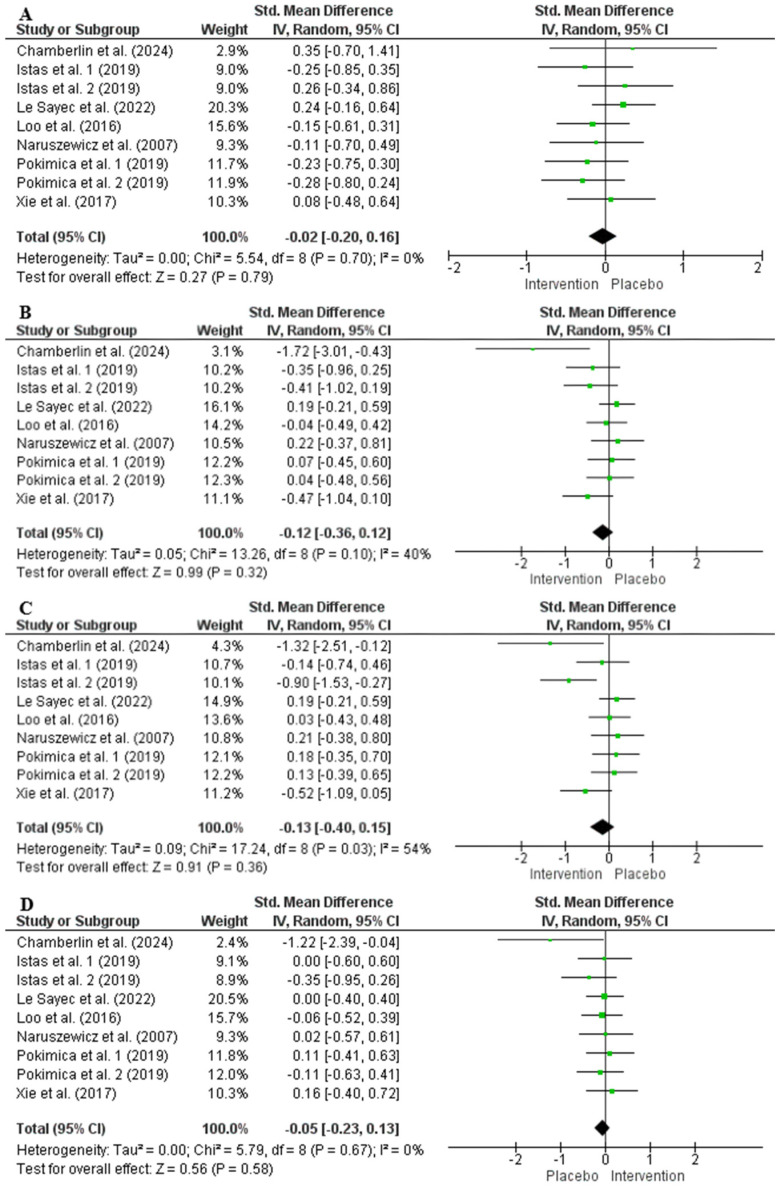
Forest plot representation of RCTs evaluating the impact of chokeberry supplementation on lipid profile [58,59,61,62,63,64,66] ((**A**): total triacylglycerols, (**B**): total cholesterol, (**C**): LDL-C, (**D**): HDL-C).

The random effects model analysis showed that chokeberry interventions did not significantly affect TAG (241 participants in the intervention group and 236 in the control group, SMD = −0.02, 95% CI: −0.20 to 0.16, *p* = 0.79, *I*^2^ = 0%), TC (SMD = −0.12, 95% CI: −0.36 to 0.12, *p* = 0.32, *I*^2^ = 40%), LDL-C (SMD = −0.13, 95% CI: −0.40 to 0.15, *p* = 0.36, *I*^2^ = 54%), or HDL-C (SMD = −0.05, 95% CI: −0.23 to 0.13, *p* = 0.58, *I*^2^ = 0%) (Figure 4). Sensitivity analysis confirmed that excluding any single study did not meaningfully influence the overall effect size for lipid profile markers. Subgroup analysis revealed that chokeberry interventions significantly reduced TC and LDL-C in participants with TC levels < 200 mg/dL, while no other significant differences were identified across different subgroups (Supplementary Table S2).

### 3.5. Impact of Chokeberry on Blood Pressure

**Figure 5:**
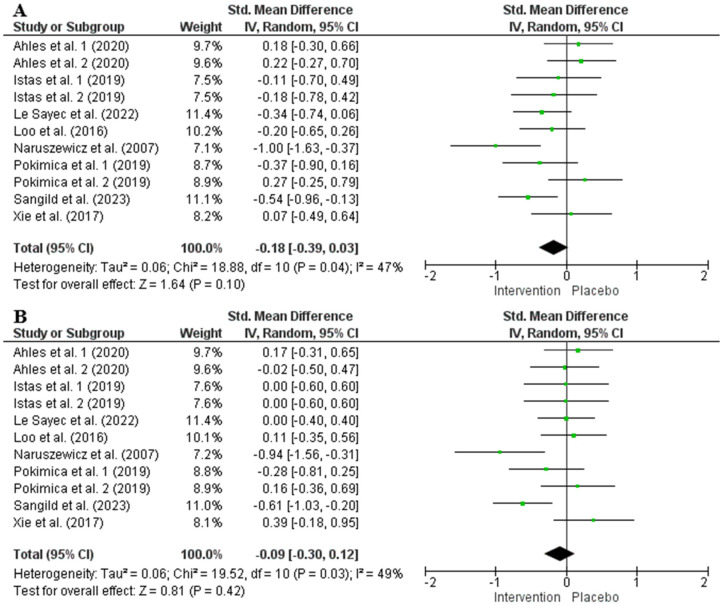
Forest plot representation of RCTs evaluating the impact of chokeberry supplementation on blood pressure [57,59,61,62,63,64,65,66] ((**A**): systolic blood pressure, (**B**): diastolic blood pressure).

The random effects model analysis indicated that chokeberry interventions had no significant effect on SBP (354 participants in the intervention group and 336 in the control group, SMD = −0.18, 95% CI: −0.39 to 0.03, *p* = 0.10, *I*^2^ = 47%) or DBP (SMD = −0.09, 95% CI: −0.30 to 0.12, *p* = 0.42, *I*^2^ = 49%) (Figure 5). Sensitivity analysis confirmed that eliminating any single study did not substantially alter the overall effect size for blood pressure. Subgroup analysis showed that chokeberry interventions significantly lowered SBP when the anthocyanin dose of interventions exceeded 50 mg/d, while no other significant differences were observed across different subgroups (Supplementary Table S2).

### 3.6. Risk of Bias, TSA, and Certainty of Evidence

The risk of bias assessment was conducted using the Cochrane risk of bias 1 (RoB 1) tool, evaluating several key methodological domains. The findings highlighted differences in study quality, with some demonstrating methodological rigor while others show potential biases that may affect the reliability of their results (Table 2). Specifically, Ahles et al. (2020) [57], Chamberlin et al. (2024) [58], Istas et al. (2019) [59], and Le Sayec et al. (2022) [61] were assessed as having a low risk of bias across all domains. In contrast, Lackner et al. (2024) [60], Naruszewicz et al. (2007) [66], Pokimica et al. (2019) [63], Sangild et al. (2023) [65], and Xie et al. (2017) [64] had an unclear risk of bias due to insufficient reporting of allocation concealment, blinding procedures, incomplete outcome data, or selective reporting. One study, Loo et al. (2016) [62], exhibited a high risk of bias, primarily due to carry-over effects, unclear allocation concealment, and a lack of blinding. The presence of a high carry-over effect suggests that residual effects from a previous intervention period may have influenced the outcomes, which is particularly concerning in crossover trials. Overall, the presence of unclear or high risk of bias in several studies underscores the importance of rigorous study design and transparent reporting in clinical research.

The TSA plots indicate that the RIS has not been reached for most outcomes and the cumulative Z-curves fail to cross the conventional or adjusted monitoring boundaries, suggesting that the available evidence is inconclusive (Supplementary Figures S1–S10). Some TSA curves show early fluctuations, but they eventually stabilize without confirming either a beneficial or a harmful effect of the intervention. The FBG TSA plot indicates that, although the required amount of data was reached, the intervention did not demonstrate a definitive effect (Supplementary Figure S4). Cumulatively, these results indicate that more RCTs are needed before drawing strong conclusions regarding the efficacy of the intervention on metabolic and cardiovascular outcomes.

The GRADE assessment indicates that the certainty of evidence for all outcomes was downgraded to very low (Table 3). The main factors contributing to these downgrades included serious risk of bias, as many studies had unclear or high RoB ratings. Additionally, serious inconsistency was noted due to high heterogeneity (*I*^2^ ≥ 50%) in some cases, while serious imprecision was observed as the 95% confidence intervals crossed zero and were moderately wide. Furthermore, the TSA results suggest that the available data were insufficient to reach firm conclusions, leading to an additional downgrade in certainty. Another major concern was the inconsistency in total baseline and/or end-of-trial (poly)phenol intake between the intervention and control groups, further complicating the interpretation of results. In the case of WC, the certainty of evidence was further downgraded due to the small number of included studies.

## 4. Discussion

The present systematic review and meta-analysis assessed the effects of chokeberry supplementation on anthropometric parameters, FBG, lipid profile, and blood pressure. The overall random effects model analysis indicated that chokeberry interventions did not significantly impact either of the assessed outcomes. However, subgroup analyses revealed that chokeberry supplementation significantly reduced TC and LDL-C in individuals with TC levels < 200 mg/dL, whereas SBP was significantly lowered when the anthocyanin dose in implemented interventions exceeded 50 mg/day; moreover, FBG was significantly increased in participants ≤ 50 years old (Supplementary Table S2). Despite these modest potential benefits (and drawbacks), the overall certainty of evidence was rated as very low due to methodological limitations, imprecision, and (poly)phenol intake inconsistency across individual studies (Table 3).

As mentioned above, this is not the first meta-analysis investigating the influence of chokeberry on cardiometabolic outcomes, as three previous meta-analyses have also explored its impact. In the first one, Rahmani et al. (2019) [32] conducted a systematic review and meta-analysis to evaluate the effects of chokeberry consumption on lipid profile, blood pressure, and inflammatory biomarkers based on seven RCTs available up to December 2018 [32]. Their findings indicated a significant increase in HDL-C and DBP following chokeberry supplementation. However, no significant effects were observed for SBP or inflammatory markers, including C-reactive protein (CRP), tumor necrosis factor (TNF-α), and interleukin-1 (IL-1). Subgroup analysis revealed that TC and LDL-C were significantly reduced in interventions lasting less than 10 weeks. Additionally, dose–response analysis suggested that TAG levels decreased significantly when chokeberry supplementation reached 300 mg/day. The overall conclusion highlighted positive effects on TC, LDL-C, and HDL-C, though with variations depending on intervention duration and dosage [32]. Moreover, in the second meta-analysis, Hawkins et al. (2021) [31] analyzed controlled trials available up to June 2020, assessing daily chokeberry supplementation on TC and blood pressure [31]. Their systematic review demonstrated that chokeberry supplementation significantly reduced TC and SBP, particularly among adults ≤50 years old. No significant effects were noted for DBP. Those authors suggested that a chokeberry supplementation is a safe and effective strategy for reducing cardiovascular risk markers, with more pronounced benefits in older adults [31]. Lastly, Christiansen et al. (2022) [33] conducted a systematic review and meta-analysis focusing on the effects of chokeberry on cardiometabolic diseases, including both RCTs and quasi-design studies [33]. This comprehensive review, which included 17 studies, found that chokeberry supplementation did not significantly affect BW, TAG, TC, HDL-C, or blood pressure. However, their quantitative analysis based on only four studies revealed a significant mean reduction in FBG by 0.44 mmol/L among participants receiving chokeberry compared with controls. Additionally, the authors noted a potential LDL-C-lowering effect with supplementation durations between 6 weeks to 3 months, although shorter interventions did not yield such effects.

However, despite some promising preliminary results, the mentioned meta-analyses have notable methodological limitations that may have affected the reliability of their findings. The meta-analysis by Rahmani et al. (2019) [32] included an RCT that focused on adolescents, even though the authors explicitly stated in the inclusion criteria section that all included studies should involve participants over 18 years old. Additionally, while the inclusion criteria also specified that only RCTs should be considered, four out of the seven included studies were non-randomized intervention trials, which introduced a high risk of bias in presented results. Similarly, the meta-analysis by Hawkins et al. (2021) [31] included an RCT that examined the effects of a juice composed of 95% citrus-based ingredients and 5% chokeberry extract, without providing a well-matched placebo. Additionally, the study in discussion did not specify which citrus fruit was used to prepare the juice, making it challenging to isolate the effects of chokeberry. This is particularly relevant as citrus fruit juices are known to contribute to improvements in cardiometabolic health [67], raising concerns about whether the observed benefits were due to chokeberry or the citrus components. Furthermore, as in the Rahmani et al. (2019) [32] meta-analysis, four out of the seven included studies in the Hawkins et al. (2021) [31] meta-analysis were non-randomized intervention trials, further questioning the reliability and strength of the reported findings. Lastly, the meta-analysis conducted by Christiansen et al. (2022) [33] included only four studies, one of which had significant methodological limitations that potentially compromised the reliability of their conclusions. Specifically, the study by Simeonov et al. (2002) [50] did not utilize an appropriately matched placebo control group, thereby increasing the risk of bias and reducing the robustness of observed effects. Moreover, the study was characterized by the inadequate reporting of key methodological details, such as randomization procedures and blinding of participants and researchers, making it challenging to assess its internal validity.

These limitations significantly impact the validity of the reported results by increasing the risk of bias and reducing the overall confidence in the findings. The inclusion of non-randomized intervention trials means that potential confounders, such as differences in baseline characteristics, diet, or lifestyle factors, may have influenced the observed effects rather than the chokeberry supplementation itself [68]. The presence of an adolescent study in the Rahmani et al. (2019) [32] analysis may also skew the results, as metabolic responses to (poly)phenols can differ between younger and older populations [69]. Similarly, in Hawkins et al. (2021) [31], the use of a multi-ingredient intervention and the lack of a properly controlled placebo could lead to an overestimation or misattribution of chokeberry’s effects [70]. Nonetheless, the small number of studies included into Christiansen et al. (2022) [33] analysis further limited the robustness and generalizability of their conclusions. Thus, even though these meta-analyses support chokeberry’s cardioprotective effects, their findings should be interpreted with caution due to the methodological weaknesses mentioned.

### 4.1. Impact of Chokeberry Supplementation on Glycemia

One of the most intriguing and unexpected findings of the meta-analysis herein relates to FBG. While the overall random effects model did not show a significant effect of chokeberry supplementation on FBG (Figure 3), subgroup analysis based on participants’ mean age revealed that FBG levels increased in individuals younger than 50 years following supplementation (Supplementary Table S2). This result contrasts with well-established evidence indicating that anthocyanins can reduce FBG levels [71]. Previous studies reported significant reductions in FBG with the supplementation of purified anthocyanins, anthocyanin-rich berries, and anthocyanin-containing foods and derivatives. These effects have been observed across various dose ranges (≤100 mg/day, 100–300 mg/day, and 300–500 mg/day) and intervention durations (4–8 weeks and 8–12 weeks) [72]. Additionally, FBG reductions have been noted when anthocyanins are consumed four times a day, particularly in individuals with baseline FBG > 100 mg/dL, and in those with insulin resistance or diabetes mellitus [72]. The glucose-lowering effects of anthocyanins are attributed to multiple mechanisms, including β-cell protection, enhanced insulin secretion, improved insulin sensitivity, better liver function, and the inhibition of carbohydrate-hydrolyzing enzymes [73]. Given these well-documented benefits, the observed increase in FBG in participants < 50 years old within the present meta-analysis suggests the presence of other underlying factors.

Indeed, as with the other observed benefits of chokeberry supplementation that will be discussed further in detail, this finding is based on a limited number of studies, specifically four trials with six study arms, involving 159 participants in the intervention group and 149 in the control group. A closer examination of these studies reveals certain limitations related to dietary background assessment, which could have influenced the results. For instance, Sangild et al. (2023) [65] did not provide any details on the background diet of the participants, while Chamberlin et al. (2024) [58] assessed participants’ habitual diets at baseline using the Healthy Eating Index (HEI) total and component scores from the self-reported Diet History Questionnaire III (DHQ III). However, no dietary evaluation was conducted at the end of the trial, making it unclear whether participants’ diets changed during the study and, if so, in what way, as variations in macronutrient distribution [74], fiber intake (total, soluble, and insoluble [75]), and other micronutrient consumption (phenolic compounds [76]) could have differentially impacted FBG levels, potentially influencing the study’s outcomes. Similarly, Pokimica et al. (2019) [63] reported no significant differences in total energy intake, macronutrient composition, or fatty acid intake between intervention groups, but only provided data for one time point (either baseline or end-of-trial, though it is unspecified). This omission makes it difficult to determine how participants’ diets evolved throughout the intervention period and if the intervention itself was responsible for the increase in FBG seen in the intervention group. In contrast, Istas et al. (2019) [59] provided a more comprehensive analysis of dietary changes during the study. However, their findings indicated differences in fiber intake between study groups (lower in the intervention group and higher in the control one), which is particularly relevant as higher dietary fiber consumption is known to lower FBG levels [75]. Additionally, variations in total (poly)phenol intake were observed among study groups (the same trend as in the case of fiber intake). Since diets rich in (poly)phenols have been shown to improve blood glucose regulation in humans by enhancing early insulin secretion and insulin sensitivity [77], these differences could have played a role in the observed changes in FBG. As a result, the inconsistencies and incomplete dietary tracking across studies represent a significant limitation, making it challenging to determine the specific impact of chokeberry supplementation on FBG. As such, dietary variations among participants, especially when unrecorded, may have contributed to the modest increase in FBG observed in the intervention groups, despite receiving chokeberry supplementation. These unaccounted dietary changes could have influenced blood glucose levels, potentially confounding the true effects of the intervention.

### 4.2. Impact of Chokeberry Supplementation on Cholesterol Levels

Regarding the lipid profile results presented in the meta-analysis herein, the reduction in TC and LDL-C observed in individuals with TC levels below 200 mg/dL following chokeberry supplementation (Supplementary Table S2) may be attributed to several mechanisms. A previous RCT demonstrated that daily supplementation with 320 mg of anthocyanins for 12 weeks can effectively lower TC and LDL-C by enhancing cholesterol efflux capacity [78], which is the initial and rate-limiting step of reverse cholesterol transport [79]. This process facilitates the removal of cholesterol from macrophages, foam cells, and atherosclerotic plaques, transporting it back to the liver for excretion as bile salts or biliary cholesterol [80]. Additionally, anthocyanin supplementation showed an inhibition of cholesteryl ester transfer protein, a plasma protein responsible for transferring cholesteryl esters from HDL-C to LDL-C, thereby leading to lower LDL-C levels [81]. Also, recent meta-analyses have further confirmed that anthocyanin-rich foods significantly reduce TC and LDL-C [71,72]. Additionally, Daneshzad et al. (2019) [82] reported that anthocyanin supplementation at doses exceeding 300 mg/day for more than 12 weeks in humans had significant lipid-lowering effects [82]. In contrast, Yang et al. (2017) reported that the optimal anthocyanin dosage for LDL-C reduction ranged between 200 and 400 mg/day, but noted that anthocyanin supplementation had no significant effect on LDL-C levels in healthy individuals [83].

As noticed, some discrepancies can be observed between reported results and those from the literature. The inconsistency between the current findings and the prior research is likely due to the limited number of studies included in the <200 mg/dL TC subgroup (only two, one of which had a small sample size of 14 participants, while the other involved two intervention arms sharing a common control group, thus increasing the probability of a false positive results). Furthermore, although previous research indicated that anthocyanin-rich foods provide lipid profile benefits when consumed at doses > 300 mg/day for over 12 weeks [82], only three of the studies included in the meta-analysis herein had a duration of 12 weeks, and just one study used chokeberry juice containing over 300 mg/day of anthocyanins, but with an 8-week duration. Consequently, the lack of significant findings in overall random effects analysis and most subgroup analyses may be attributed to the limited number of studies, short duration of interventions, and the insufficient anthocyanin content in the evaluated extracts, powders, or juices, as anthocyanins may be the key (poly)phenol subclass responsible for the cardiometabolic benefits of chokeberry consumption [17,31]. Moreover, the positive findings in the <200 mg/dL TC subgroup may represent false positive results, particularly when considered alongside the TSA findings, which suggest insufficient evidence to confirm a true effect.

### 4.3. Impact of Chokeberry Supplementation on Systolic Blood Pressure and Body Weight

Although blood pressure is a significant cardiometabolic risk factor, chokeberry consumption may not have a meaningful impact on its regulation, as assessed by the overall random effects model (Figure 5). Subgroup analysis suggested that only interventions providing more than 50 mg/day of anthocyanins showed potential benefits in lowering SBP (Supplementary Table S2); however, this finding was based on just four studies with 137 and 131 participants in the intervention and control groups, respectively, limiting its reliability. The existing literature also supports inconsistent effects of anthocyanin supplementation on blood pressure. For instance, Pan et al. (2025) [72] reported no significant impact on SBP or DBP, though subgroup analysis indicated an increase in SBP with anthocyanin doses below 100 mg/day, particularly in overweight and obese individuals. In contrast, anthocyanin supplementation reduced SBP in individuals diagnosed with dyslipidemia or in trials lasting more than 12 weeks [72]. Another meta-analysis concluded that anthocyanin supplementation had no significant effects on either SBP or DBP, regardless of study design, duration, dosage, population characteristics, or participant demographics [82]. Similar to the findings for TC and LDL-C, the observed SBP benefits of chokeberry supplementation in individuals under 50 years old may be a false positive due to the limited number of included studies and small sample sizes.

Furthermore, blood pressure reduction is often linked to weight loss. Yang et al. (2023) [84] found that SBP and DBP decreased by 5.79 mmHg and 3.36 mmHg, respectively, following a mean BMI reduction of 2.27 kg/m^2^, while a greater BMI reduction of 4.12 kg/m^2^ led to SBP and DBP reductions of 6.65 mmHg and 3.63 mmHg, respectively. Additionally, the American College of Cardiology/American Heart Association reported that even modest weight loss can lead to significant blood pressure reductions, with each kilogram of weight loss being associated with a 1 mmHg decrease in SBP [85]. However, none of the trials assessing blood pressure effects of chokeberry supplementation (and none of the trials included in the meta-analysis herein that assessed body weight, BMI, or waist circumference [Figure 2]) reported any weight loss, including those that found a positive impact on SBP with anthocyanin doses exceeding 50 mg/day. Indeed, it was shown that anthocyanin supplementation did not decrease body weight, BMI, or waist circumference independently of a caloric deficit [82]. This suggests that any observed reductions in blood pressure were not influenced by changes in body weight or interventions alone, being influenced, most probably, by other underlying methodological factors.

### 4.4. Chokeberry Proanthocyanidins and Evaluated Cardiometabolic Outcomes

Nonetheless, proanthocyanidins are another class of bioactive compounds found in chokeberry interventions. A meta-analysis of human studies showed proanthocyanidin supplementation significantly reduced TAG and increased recombinant apolipoprotein A1. Subgroup analysis indicated that TG levels decreased in older adults (≥60 years), while TC was reduced in individuals with a BMI < 24. Interventions lasting over 8 weeks led to reductions in TG and LDL-C and an increase in HDL-C, suggesting a potential role for proanthocyanidins in lipid metabolism regulation [86]. In another meta-analysis, Ren et al. (2021) [87] showed that proanthocyanidins significantly reduced SBP and DBP. Subgroup analysis showed that SBP reductions were more evident in studies lasting <12 weeks, while DBP reductions were significant in trials lasting ≥12 weeks. Additionally, SBP decreased more in individuals with a BMI ≥25, whereas DBP was more affected in those with a BMI <25. Notably, low-dose PCs (<245 mg/day) effectively lowered SBP and DBP, although meta-regression analysis found no dose-dependent effect on blood pressure [87].

Chokeberries have a total proanthocyanidin content of approx. 1500 mg (−)epicatechin equivalents/100 g fresh weight [88]. Although the dosage of proanthocyanidins in the study interventions included in the meta-analysis herein varied from 3.3 mg/day [59] to 576 mg/day [62], their direct impact on cardiometabolic health outcomes may be limited due to, as already mentioned, extremely low bioavailability, which restricts their systemic effects [89]. Rather than being efficiently absorbed in the gastrointestinal tract, proanthocyanidins primarily exert their effects within the gut, where they influence digestive enzyme activity, gut microbiota composition, and intestinal barrier integrity, rather than directly modulating metabolic pathways in target organs [90,91,92]. This limited bioavailability not only affected their direct physiological impact but also complicated their independent assessment in the present analysis. As proanthocyanidins were intrinsically linked with anthocyanins in the included chokeberry interventions, the concentrations of both compounds were proportional, making it challenging to distinguish their specific effects. Consequently, a separate subgroup analysis for proanthocyanidins was not conducted, as it would have merely replicated the findings observed for anthocyanins.

### 4.5. (Poly)phenol Intake Inconsistency

A key limitation observed in almost all of the included studies was the insufficient monitoring of (poly)phenol intake both pre- and post-intervention, considering that dietary (poly)phenols are closely associated with various cardiometabolic benefits. It is known that dietary measurement is a major problem in nutrition science [93]. Evidence from large cohort studies has highlighted that even modest increases in (poly)phenol intake (100–200 mg/day) are significantly associated with reductions in metabolic syndrome risk and improvements in lipid profiles, blood pressure, and inflammatory markers [94,95]. Although it is methodologically challenging, indirect approaches such as food diaries, dietary recalls, and biomarker analyses (e.g., plasma or urinary polyphenol levels) are commonly used to assess (poly)phenol intake and compliance. However, such methods were inconsistently applied across the included studies, limiting the reliability of dietary intake estimations. In the studies that assessed total (poly)phenol intake before and after the intervention (Loo et al., 2016 [62]; Istas et al., 2019 [59]; Le Sayec et al., 2022 [61]; Lackner et al., 2024 [60]), it is unclear if the (poly)phenol content of the interventions was accounted for in the reported post-intervention (poly)phenol intake values. For example, in the trial conducted by Loo et al. (2016) [62], the intervention included 300 mL/day of chokeberry juice (providing 966 mg anthocyanins, 576 mg proanthocyanidins, 351 mg phenolic acids, and 55 mg flavonoids) and 3 g/day of chokeberry powder (containing 169 mg proanthocyanidins, 58 mg anthocyanins, 16 mg phenolic acids, and 3.3 mg flavonoids). However, it is unclear why the (poly)phenol content of this intervention was not accounted for in the reported post-intervention (poly)phenol intake, making it challenging to accurately assess the effectiveness of the supplementation. Furthermore, the same studies paradoxically reported that the post-intervention (poly)phenol intake of participants decreased, despite the administration of (poly)phenol-rich interventions. Istas et al. (2019) [59] reported a significant change in (poly)phenol intake between groups, where the intervention group experienced a decrease, potentially reducing the efficacy of the supplementation, while the control group showed an increase. Lackner et al. (2024) [60] similarly found a significantly higher (poly)phenol intake in the placebo group compared with the intervention group, which may have confounded the results. Le Sayec et al. (2022) [61] reported marginally significant differences in (poly)phenol intake between groups; however, as discussed, other unrecognized dietary factors might have influenced the reported results [96]. This raises concerns about whether participants’ dietary adherence, reporting accuracy, or potential dietary compensations influenced the measured (poly)phenol intake.

Moreover, studies such as the ones by Sangild et al. (2023) [65], Ahles et al. (2020) [57], and Chamberlin et al. (2024) [58] provided dietary guidelines restricting high-(poly)phenol foods but did not objectively measure adherence. Although Chamberlin et al. (2024) [58] assessed baseline dietary habits through HEI, they did not specifically quantified (poly)phenol intake, making it unclear whether intake remained constant throughout the study. HEI is a useful tool for assessing overall diet quality, but it has several limitations when it comes to accurately evaluating (poly)phenol intake [97]. Similarly, Naruszewicz et al. (2007) [66] relied on participants’ self-reported adherence without implementing objective methods to quantify (poly)phenol intake or potential dietary changes.

A further limitation pertains to the selective reporting of (poly)phenol subtypes, which may lead to an incomplete understanding of dietary effects. For example, Loo et al. (2016) [62] reported changes in phenolic acids and quercetin intake but omitted data on other (poly)phenol groups, which were of the most interest for the purpose of their study, thus missing crucial dietary interactions. Pokimica et al. (2019) [63] assessed habitual dietary intake through food frequency questionnaires and 24 h recalls, yet failed to report (poly)phenol intake levels at baseline and post-intervention. Additionally, Xie et al. (2017) [64] provided a nutrient intake table but did not clarify whether reported values represented baseline, post-intervention, or an average across the study period, making it difficult to determine whether dietary intake remained stable or changed other the duration of the trial. 

Consequently, the lack of a clear and consistent assessment of total (poly)phenol intake in the included studies complicates the interpretation of results, making it difficult to determine the true impact of chokeberry supplementation on cardiometabolic health outcomes. Without a comprehensive assessment of all (poly)phenol subtypes and clear reporting on dietary changes, the validity of chokeberry RCT supplementation studies remain questionable. These limitations emphasize the need for future studies to incorporate direct (poly)phenol intake measurements and standardized dietary tracking methods to improve the reliability of findings.

### 4.6. Randomized Versus Non-Randomized Intervention Trials of Chokeberry Supplementation

Non-randomized intervention trials have also been allegedly cited as evidence supporting the benefits of chokeberry supplementation. For instance, Broncel et al. (2010) [28] conducted a study in which patients with metabolic syndrome received a daily dose of 300 mg of chokeberry extract over a two-month period. This intervention led to significant reductions in SBP, DBP, TAG, TC, and LDL-C. Similarly, Sikora et al. (2012) [42] reported that supplementation with the same extract in patients with metabolic syndrome resulted in notable decreases in TAG, TC, and LDL-C, while no significant changes were observed in HDL-C, BMI, or WC. The same research group further demonstrated that a two-month administration of a chokeberry preparation significantly lowered TC, LDL-C, and SBP in other individuals with metabolic syndrome [45].

Furthermore, Kardum et al. (2015) [46] investigated the impact of (poly)phenol-rich chokeberry juice on 24 h ambulatory blood pressure in individuals with untreated high-normal BP or grade I hypertension. By the end of the intervention, significant reductions were observed in 24 h and daytime systolic and diastolic BP, along with a notable decrease in TAG levels. Milutinović et al. (2019) [47] assessed the impact of three months of chokeberry juice supplementation in patients with type 2 diabetes, observing a significant reduction in LDL-C levels post-intervention. Lastly, Tasic et al. (2021) [30] evaluated the effects of a four-week supplementation with a standardized *A. melanocarpa* extract on clinical and biochemical parameters in patients with metabolic syndrome. The intervention significantly influenced SBP and DBP. After two weeks, TC levels were significantly reduced in female participants with metabolic syndrome and diabetes, while LDL-C levels significantly decreased in the female metabolic syndrome group. In diabetic groups, TAG levels were significantly lower after four weeks, whereas no significant changes were observed in non-diabetic participants. Overall, an increased intake of (poly)phenols or chokeberry extract supplementation was associated with beneficial effects on cholesterol levels, blood pressure, and glycemic control in non-randomized intervention trials.

These trials, while providing valuable preliminary insights, inherently carry a higher risk of bias compared with RCTs (which were included in the meta-analysis herein) [98]. The primary limitation of non-randomized intervention trials is their inability to eliminate selection bias, as participant assignment is not random and may be influenced by underlying health statuses, lifestyle factors, or other confounders [99]. This can lead to systematic differences between intervention and control groups, making it difficult to attribute observed effects solely to the supplementation itself. Additionally, non-randomized intervention trials often lack strict control over external variables such as dietary intake, physical activity, and medication use, all of which can significantly impact cardiometabolic markers [93]. Without adequate control mechanisms, improvements in lipid profiles, blood pressure, or glycemic markers may be erroneously linked to chokeberry supplementation rather than to underlying differences in participant characteristics or concurrent lifestyle modifications.

Another major concern with non-randomized intervention trials is the increased likelihood of false positive results, where beneficial effects are overstated due to uncontrolled confounding factors and inadequate blinding [98]. When evaluating supplementation protocols, such as chokeberry interventions, studies lacking randomization and placebo controls may suffer from observer bias and the placebo effect, leading to exaggerated claims of efficacy. Moreover, small sample sizes and short intervention durations, which are common in non-randomized trials, can inflate effect sizes due to chance variations rather than genuine physiological benefits, creating a misleading perception of effectiveness of chokeberry supplementation. Therefore, while non-randomized intervention trials can serve as a foundation for hypothesis generation regarding chokeberry supplementation, their findings must be interpreted with caution and not be over generalized.

### 4.7. Meta-Analysis Limitations and Future Perspectives

Despite the comprehensive approach employed in this meta-analysis, several limitations must be acknowledged. First, the small number of eligible RCTs and some variability in participant characteristics and intervention types contributed to the lack of results and assessment of publication bias. The differences in chokeberry supplementation forms (extracts, powders, and juices), quantity (90 mg/day to 300 mL/day), anthocyanin content (3.6 to 966 mg/day), and intervention durations (4 to 24 weeks) made direct comparisons somehow challenging and may have influenced the reported effects on cardiometabolic markers (though subgroup analyses based on these differences were performed). Second, the certainty of evidence was rated as very low due to methodological limitations, including unclear or high risk of bias in several trials, imprecision in outcome reporting, and inconsistencies in baseline and post-intervention (poly)phenol intake across studies. The lack of standardized dietary assessments further complicated the interpretation of results, as background diet could have confounded the metabolic effects of chokeberry supplementation. Third, the TSA indicated that the RIS was not met for most outcomes, suggesting that the available data remained insufficient to draw definitive conclusions. This underscores the need for additional high-quality RCTs with larger sample sizes, longer intervention durations, and well-matched placebo controls to establish more conclusive evidence.

Notably, none of the included trials used fresh chokeberry as an intervention, likely due to its astringent and tart taste, which may affect participant compliance. The absence of whole fruit supplementation limits our understanding of how the synergistic action of its fiber and (poly)phenol matrix impacts cardiometabolic health [100], as processing methods can alter bioavailability and overall efficacy of extracts [101]. Future research could consider investigating the health effects of fresh chokeberry consumption, as whole food interventions may offer distinct metabolic benefits compared with processed derivatives.

That being said, future research should focus on well-powered rigorously designed RCTs that ensure standardized chokeberry interventions, using well-characterized interventions with known bioactive compound content. Additionally, integrating objective pre- and post-intervention dietary assessments, such as food diaries or biomarkers of (poly)phenol intake, will help clarify the independent effects of chokeberry supplementation. Finally, future studies should assess the long-term effects of chokeberry supplementation (>12 weeks), particularly in populations with pre-existing cardiometabolic disorders, to determine its clinical relevance for reducing risk and managing the diseases. By addressing these gaps, future research can provide stronger evidence on the potential benefits of chokeberry supplementation in cardiometabolic health.

## 5. Conclusions

This systematic review and meta-analysis critically analyzed and evaluated available RCTs on chokeberry supplementation by applying a rigorous methodological framework and advanced tools for bias and certainty assessment. It was found that chokeberry supplementation did not significantly improve body weight, glycemic control, lipid profile, or blood pressure in the general adult population. While subgroup analyses suggested potential reductions in total cholesterol, LDL-C, and systolic blood pressure in specific conditions, and an increase in fasting blood glucose, these findings were based on limited and low-certainty evidence. Risk of bias, inconsistencies in dietary (poly)phenol intake, and methodological limitations across the included studies further weakened the reliability of the results. Given the inconclusive nature of the current evidence, future well-designed larger-scale RCTs with standardized interventions and rigorous dietary assessments are needed to clarify the potential cardiometabolic benefits of chokeberry supplementation.

## Figures and Tables

**Table 2:** Risk of bias assessment according to the Cochrane collaboration risk of bias assessment tool.

Study, Year [Reference]	Random Sequence Generation	Carry-Over Effect	Allocation Concealment	Blinding of Participants and Personnel	Blinding of Outcome Assessment	Incomplete Outcome Data	Selective Reporting	Overall Assessment of Risk of Bias
Ahles et al. (2020) [57]	Low	-	Low	Low	Low	Low	Low	Low
Chamberlin et al. (2024) [58]	Low	-	Low	Low	Low	Low	Low	Low
Istas et al. (2019) [59]	Low	-	Low	Low	Low	Low	Low	Low
Lackner et al. (2024) [60]	Low	-	Unclear	Unclear	Unclear	Low	Low	Unclear
Le Sayec et al. (2022) [61]	Low	-	Low	Low	Low	Low	Low	Low
Loo et al. (2016) [62]	Low	High	Unclear	Unclear	Unclear	Low	Low	High
Naruszewicz et al. (2007) [66]	Unclear	-	Unclear	Low	Low	Low	Low	Unclear
Pokimica et al. (2019) [63]	Low	-	Unclear	Low	Low	Low	Low	Unclear
Sangild et al. (2023) [65]	Unclear	Low	Low	Low	Low	Unclear	Unclear	Unclear
Xie et al. (2017) [64]	Low	-	Unclear	Low	Low	Low	Low	Unclear

**Table 3:** The grading of recommendations assessment, development, and evaluation (GRADE) framework used to assess the quality of evidence for each outcome.

No of Studies	Certainty Assessment						No. of Patients		Effect	Certainty
	Design	Risk of Bias	Inconsistency	Indirectness	Imprecision	Other Considerations	Treatment Group	Control Group	SMD (95% CI)	
**BW**										
5	RCTs	Serious ^a^	Not serious	Not serious	Very serious ^c,d^	(Poly)phenol intake inconsistency ^e^	176	167	0.01 [−0.20, 0.22]	⨁◯◯◯ Very low
**BMI**										
5	RCTs	Serious ^a^	Not serious	Not serious	Very serious ^c,d^	(Poly)phenol intake inconsistency ^e^	188	189	0.03 [−0.17, 0.23]	⨁◯◯◯ Very low
**WC**										
3	RCTs	Serious ^a^	Not serious	Not serious	Very serious ^c,d^	(Poly)phenol intake inconsistency ^e^ Small number of included studies ^f^	99	100	0.02 [−0.26, 0.30]	⨁◯◯◯ Very low
**TAG**										
7	RCTs	Serious ^a^	Not serious	Not serious	Very serious ^c,d^	(Poly)phenol intake inconsistency ^e^	241	236	−0.02 [−0.20, 0.16]	⨁◯◯◯ Very low
**TC**										
7	RCTs	Serious ^a^	Not serious	Not serious	Very serious ^c,d^	(Poly)phenol intake inconsistency ^e^	241	236	−0.12 [−0.36, 0.12]	⨁◯◯◯ Very low
**LDL-C**										
7	RCTs	Serious ^a^	Serious ^b^	Not serious	Very serious ^c,d^	(Poly)phenol intake inconsistency ^e^	241	236	−0.13 [−0.40, 0.15]	⨁◯◯◯ Very low
**HDL-C**										
7	RCTs	Serious ^a^	Not serious	Not serious	Very serious ^c,d^	(Poly)phenol intake inconsistency ^e^	241	236	−0.05 [−0.23, 0.13]	⨁◯◯◯ Very low
**FBG**										
7	RCTs	Serious ^a^	Not serious	Not serious	Very serious ^c,d^	(Poly)phenol intake inconsistency ^e^	267	256	0.21 [−0.01, 0.43]	⨁◯◯◯ Very low
**SBP**										
8	RCTs	Serious ^a^	Not serious	Not serious	Very serious ^c,d^	(Poly)phenol intake inconsistency ^e^	354	336	−0.18 [−0.39, 0.03]	⨁◯◯◯ Very low
**DBP**										
8	RCTs	Serious ^a^	Not serious	Not serious	Very serious ^c,d^	(Poly)phenol intake inconsistency ^e^	354	336	−0.09 [−0.30, 0.12]	⨁◯◯◯ Very low

^a^. Serious concerns regarding the risk of bias since most of the studies were rated as unclear or high risk of bias. Downgraded by one level. ^b^. Serious inconsistency since *I*^2^ ≥ 50%. Downgraded by one level. ^c^. Serious imprecision since the 95% CI crossed zero and was moderately wide. Downgraded by one level. ^d^. According to TSA results, the evidence was inconclusive, more data are needed before drawing a strong conclusion about the effect of the intervention. Downgraded by one level. ^e^. Total baseline and/or end-of-trial (poly)phenol intakes were not measured or there were differences between intervention and control groups regarding (poly)phenol intake at one measuring point. Downgraded by one level. ^f^. A small number of included studies assessed the outcome. Downgraded by one level. Abbreviations: standardized mean difference (SMD), confidence interval (CI), randomized controlled trials (RCTs), body weight (BW), body mass index (BMI), waist circumference (WC), triacylglycerol (TAG), total cholesterol (TC), low-density lipoprotein cholesterol (LDL-C), high-density lipoprotein cholesterol (HDL-C), fasting blood glucose (FBG), systolic blood pressure (SBP), diastolic blood pressure (DBP).

## Data Availability

The original contributions presented in this study are included in this article/Supplementary Material. Further inquiries can be directed to the corresponding author(s).

## Supplementary Materials

The following supporting information can be downloaded at: https://www.mdpi.com/article/10.3390/nu17091488/s1, Supplementary Table S1. Search strategies including the key terms and the queries for each database; Supplementary Table S2. Result of subgroup analysis of included studies in the meta-analysis; Supplementary Figures S1–S10. Trial sequential analysis evaluating the robustness of the cumulative evidence for body weight, body mass index, waist circumference, fasting blood glucose, triacylglycerol, total cholesterol, LDL-C, HDL-C, SBP, and DBP, respectively.
