# Supplementary material for: Impact of a Chokeberry (Aronia melanocarpa (Michx.) Elliott) Supplementation on Cardiometabolic Outcomes: A Critical Systematic Review and Meta-Analysis of Randomized Controlled Trials
Source: Nutrients. 2025 Apr 28;17(9):1488. doi: 10.3390/nu17091488 (PMC12073822; doi:10.3390/nu17091488)
Supplement: Supplementary file 1 [file nutrients-17-01488-s001.zip › nutrients-3592181-supplementary.pdf]

Supplementary Table S1. Search strategies including the key terms and the queries for each database.

Database	Key terms and the queries
PubMed	(("photinia"[MeSH Terms] OR "photinia"[All Fields] OR "aronia"[All Fields] OR "melanocarpa"[All Fields] OR "chokeberr*"[All Fields] OR "Aronia melanocarpa"[All Fields] OR "black chokeberry"[All Fields]) AND ("randomized controlled trial"[All Fields] OR "randomised controlled trial"[All Fields] OR "random*"[All Fields] OR "RCT"[All Fields] OR "clinical trial"[All Fields] OR "controlled trial"[All Fields] OR "trial*"[All Fields] OR "placebo-controlled"[All Fields] OR "double-blind"[All Fields] OR "single-blind"[All Fields] OR "blind*"[All Fields])) NOT ("review"[Publication Type] OR "review literature as topic"[MeSH Terms] OR "review"[All Fields])
WebOfScience	ALL=((aronia OR melanocarpa OR chokeberr* OR "Aronia melanocarpa" OR "black chokeberry") AND ("randomized controlled trial" OR "randomised controlled trial" OR random* OR "RCT" OR "clinical trial" OR "controlled trial" OR trial* OR "placebo-controlled" OR "double-blind" OR "single-blind" OR blind*) NOT review)
Scopus	(TITLE-ABS-KEY ("randomized controlled trial" OR "randomised controlled trial" OR random* OR "RCT" OR "clinical trial" OR "controlled trial" OR trial* OR "placebo-controlled" OR "double-blind" OR "single-blind" OR blind*) AND TITLE-ABS-KEY (aronia OR melanocarpa OR chokeberr* OR "Aronia melanocarpa" OR "black chokeberry") AND NOT TITLE-ABS-KEY (review))

Supplementary Table S2. Result of subgroup analysis of included studies in the meta-analysis.

Sub-grouped by	No. of trials	Effect size[1]	95% CI, P value	I^2 (%)	P for heterogeneity	P for between subgroup heterogeneity
**BW (All trials)**	**6**	**0.01**	**[-0.20, 0.22], 0.94**	**0**	**0.97**	
Participants health status						0.59
Healthy subjects	3	-0.07	[-0.42, 0.28], 0.70	0	0.89	
Cardiometabolic disease subjects	3	0.05	[-0.21, 0.32], 0.70	0	0.81	
Intervention type						0.94
Extract and/or powder	4	0.00	[-0.26, 0.26], 0.98	0	0.82	
Juice	2	0.02	[-0.35, 0.39], 0.91	0	0.88	
Anthocyanins dosage						0.94
≤50 mg/d	4	0.00	[-0.26, 0.26], 0.98	0	0.82	
>50 mg/d	2	0.02	[-0.35, 0.39], 0.91	0	0.88	
Trial duration						0.94
≤8 wk	2	0.02	[-0.35, 0.39], 0.91	0	0.88	
>8 wk	4	0.00	[-0.26, 0.26], 0.98	0	0.82	
Participants age						0.97
≤50 years old	4	0.01	[-0.29, 0.31], 0.94	0	0.82	
>50 years old	2	0.01	[-0.29, 0.30], 0.97	0	0.98	
**BMI (All trials)**	**6**	**0.03**	**[-0.17, 0.23], 0.78**	**0**	**0.76**	
Intervention type						0.37
Extract and/or powder	3	0.12	[-0.16, 0.41], 0.41	0	0.56	
Juice	3	-0.07	[-0.35, 0.22], 0.65	0	0.72	
Anthocyanins dosage						0.36
≤50 mg/d	3	0.12	[-0.16, 0.39], 0.41	0	0.55	
>50 mg/d	3	-0.07	[-0.37, 0.22], 0.63	0	0.74	
Trial duration						0.35
≤8 wk	4	-0.05	[-0.31, 0.21], 0.71	0	0.87	
>8 wk	2	0.15	[-0.18, 0.49], 0.37	4	0.31	
Participants age						0.91
≤50 years old	3	0.04	[-0.31, 0.40], 0.81	24	0.27	
>50 years old	3	0.02	[-0.25, 0.29], 0.89	0	0.99	
**TAG (All trials)**	**9**	**-0.02**	**[-0.20, 0.16], 0.79**	**0**	**0.70**	
Participants health status						0.66
<200 mg/dL	3	0.05	[-0.34, 0.45], 0.79	0	0.42	
≥200 mg/dL	6	-0.05	[-0.25, 0.16], 0.66	0	0.61	
Intervention type						0.17
Extract and/or powder	5	0.08	[-0.15, 0.32], 0.49	0	0.65	
Juice	4	-0.17	[-0.45, 0.10], 0.22	0	0.76	
Anthocyanins dosage						0.37
≤50 mg/d	5	0.04	[-0.19, 0.27], 0.73	0	0.43	
>50 mg/d	4	-0.13	[-0.41, 0.16], 0.39	0	0.81	
Trial duration						0.13
≤8 wk	5	-0.16	[-0.41, 0.09], 0.21	0	0.88	
>8 wk	4	0.12	[-0.14, 0.38], 0.37	0	0.57	
Participants age						0.56
≤50 years old	6	-0.07	[-0.32, 0.17], 0.55	0	0.65	

>50 years old	3	0.03	[-0.23, 0.30], 0.80	0	0.39	
**TC (All trials)**	**9**	**-0.12**	**[-0.36, 0.12], 0.32**	**40**	**0.10**	
Participants health status						0.05
<200 mg/dL	3	-0.61	[-1.20, -0.01], 0.05	47	0.15	
≥200 mg/dL	6	0.02	[-0.18, 0.23], 0.81	0	0.54	
Intervention type						0.95
Extract and/or powder	5	-0.13	[-0.44, 0.18], 0.41	38	0.17	
Juice	4	-0.15	[-0.60, 0.30], 0.52	56	0.08	
Anthocyanins dosage						0.93
≤50 mg/d	5	-0.15	[-0.43, 0.13], 0.30	29	0.23	
>50 mg/d	4	-0.12	[-0.61, 0.37], 0.63	60	0.06	
Trial duration						0.55
≤8 wk	5	-0.05	[-0.42, 0.31], 0.77	47	0.11	
>8 wk	4	-0.21	[-0.56, 0.14], 0.24	42	0.16	
Participants age						0.06
≤50 years old	6	-0.30	[-0.63, 0.04], 0.08	42	0.13	
>50 years old	3	0.12	[-0.15, 0.39], 0.39	0	0.71	
**LDL-C (All trials)**	**9**	**-0.13**	**[-0.40, 0.15], 0.36**	**54**	**0.03**	
Participants health status						0.04
<200 mg/dL	3	-0.67	[-1.33, -0.01], 0.05	56	0.10	
≥200 mg/dL	6	0.06	[-0.14, 0.26], 0.57	0	0.43	
Intervention type						0.53
Extract and/or powder	5	-0.20	[-0.61, 0.21], 0.33	64	0.03	
Juice	4	-0.02	[-0.42, 0.37], 0.91	44	0.15	
Anthocyanins dosage						0.50
≤50 mg/d	5	-0.21	[-0.60, 0.18], 0.30	63	0.03	
>50 mg/d	4	-0.01	[-0.43, 0.40], 0.95	46	0.14	
Trial duration						0.24
≤8 wk	5	0.04	[-0.27, 0.35], 0.80	29	0.23	
>8 wk	4	-0.31	[-0.79, 0.18], 0.22	69	0.02	
Participants age						0.07
≤50 years old	6	-0.32	[-0.73, 0.09], 0.12	61	0.03	
>50 years old	3	0.14	[-0.13, 0.41], 0.31	0	0.85	
**HDL-C (All trial)**	**9**	**-0.05**	**[-0.23, 0.13], 0.58**	**0**	**0.67**	
Participants health status						0.22
<200 mg/dL	3	-0.35	[-0.90, 0.19], 0.21	40	0.19	
≥200 mg/dL	6	0.01	[-0.19, 0.21], 0.92	0	0.98	
Intervention type						0.66
Extract and/or powder	5	-0.02	[-0.26, 0.22], 0.86	0	0.82	
Juice	4	-0.11	[-0.45, 0.22], 0.51	27	0.25	
Anthocyanins dosage						0.85
≤50 mg/d	5	-0.05	[-0.28, 0.19], 0.70	0	0.70	
>50 mg/d	4	-0.09	[-0.44, 0.27], 0.64	29	0.64	
Trial duration						0.81
≤8 wk	5	-0.07	[-0.34, 0.19], 0.57	5	0.38	
>8 wk	4	-0.03	[-0.29, 0.23], 0.82	0	0.68	
Participants age						0.72
≤50 years old	6	-0.09	[-0.35, 0.18], 0.52	11	0.35	
>50 years old	3	-0.02	[-0.29, 0.25], 0.90	0	0.97	
**FBG (All trial)**	**9**	**0.21**	**[-0.01, 0.43], 0.06**	**34**	**0.14**	

Participants health status						0.20
Healthy subjects	3	0.71	[-0.16, 1.59], 0.11	72	0.03	
Cardiometabolic disease subjects	6	0.13	[-0.06, 0.32], 0.18	0	0.69	
Intervention type						0.26
Extract and/or powder	5	0.12	[-0.10, 0.34], 0.29	0	0.69	
Juice	4	0.45	[-0.08, 0.98], 0.10	66	0.03	
Anthocyanins dosage						0.62
≤50 mg/d	4	0.16	[-0.09, 0.42], 0.21	0	0.78	
>50 mg/d	5	0.29	[-0.14, 0.71], 0.19	64	0.03	
Trial duration						0.60
≤8 wk	5	0.30	[-0.15, 0.76], 0.19	64	0.03	
>8 wk	4	0.17	[-0.07, 0.40], 0.17	0	0.78	
Participants age						0.14
≤50 years old	6	0.37	[0.07, 0.67], 0.02	37	0.16	
>50 years old	3	-0.01	[-0.28, 0.25], 0.91	0	0.79	
**SBP (All trial)**	**11**	**-0.18**	**[-0.39, 0.03], 0.10**	**47**	**0.04**	
Participants health status						0.51
Normal BP	3	-0.06	[-0.40, 0.28], 0.72	0	0.82	
Prehypertension	8	-0.21	[-0.48, 0.06], 0.13	61	0.01	
Intervention type						0.66
Extract and/or powder	8	-0.20	[-0.47, 0.06], 0.14	55	0.03	
Juice	3	-0.10	[-0.46, 0.26], 0.57	36	0.21	
Anthocyanins dosage						0.007
≤50 mg/d	7	0.00	[-0.19, 0.19], 0.99	0	0.48	
>50 mg/d	4	-0.49	[-0.78, -0.19], 0.001	30	0.23	
Trial duration						0.51
≤8 wk	4	-0.30	[-0.77, 0.17], 0.21	68	0.02	
>8 wk	7	-0.12	[-0.35, 0.10], 0.29	33	0.18	
Participants age						0.89
≤50 years old	6	-0.17	[-0.43, 0.09], 0.20	30	0.21	
>50 years old	5	-0.20	[-0.57, 0.17], 0.29	66	0.02	
**DBP (All trial)**	**11**	**-0.09**	**[-0.30, 0.12], 0.42**	**49**	**0.03**	
Participants health status						0.17
Normal BP	3	0.14	[-0.20, 0.48], 0.42	0	0.57	
Prehypertension	8	-0.16	[-0.42, 0.10], 0.23	57	0.02	
Intervention type						0.52
Extract and/or powder	8	-0.12	[-0.41, 0.16], 0.39	59	0.02	
Juice	3	0.01	[-0.28, 0.30], 0.95	0	0.43	
Anthocyanins dosage						0.04
≤50 mg/d	7	0.09	[-0.10, 0.28], 0.36	0	0.94	
>50 mg/d	4	-0.41	[-0.83, 0.02], 0.06	66	0.03	
Trial duration						0.52
≤8 wk	4	-0.21	[-0.66, 0.25], 0.37	67	0.03	
>8 wk	7	-0.04	[-0.28, 0.21], 0.77	41	0.12	
Participants age						0.98
≤50 years old	6	-0.09	[-0.40, 0.23], 0.59	51	0.07	
>50 years old	5	-0.09	[-0.41, 0.23], 0.58	56	0.06	

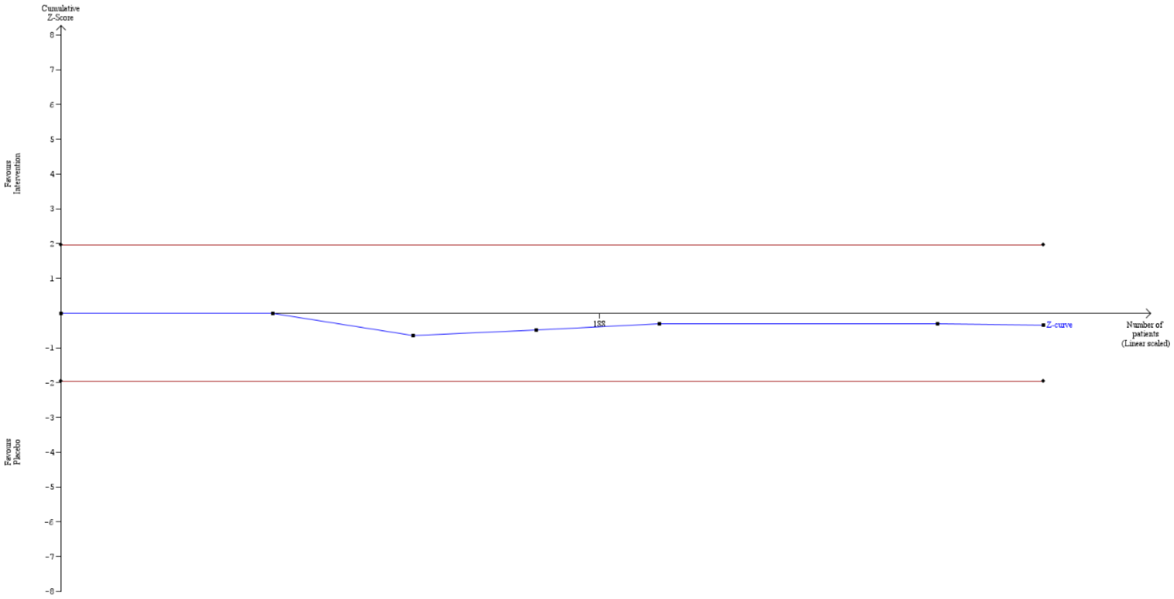

Supplementary Figure S1. Trial sequential analysis evaluating the robustness of the cumulative evidence for body weight.

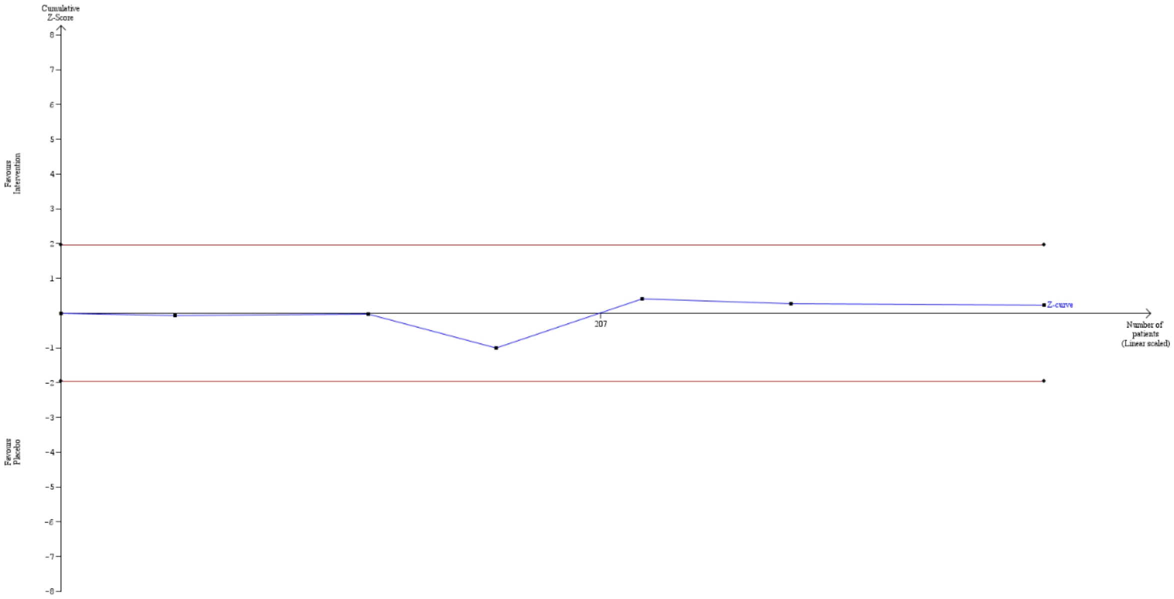

Supplementary Figure S2. Trial sequential analysis evaluating the robustness of the cumulative evidence for body mass index.

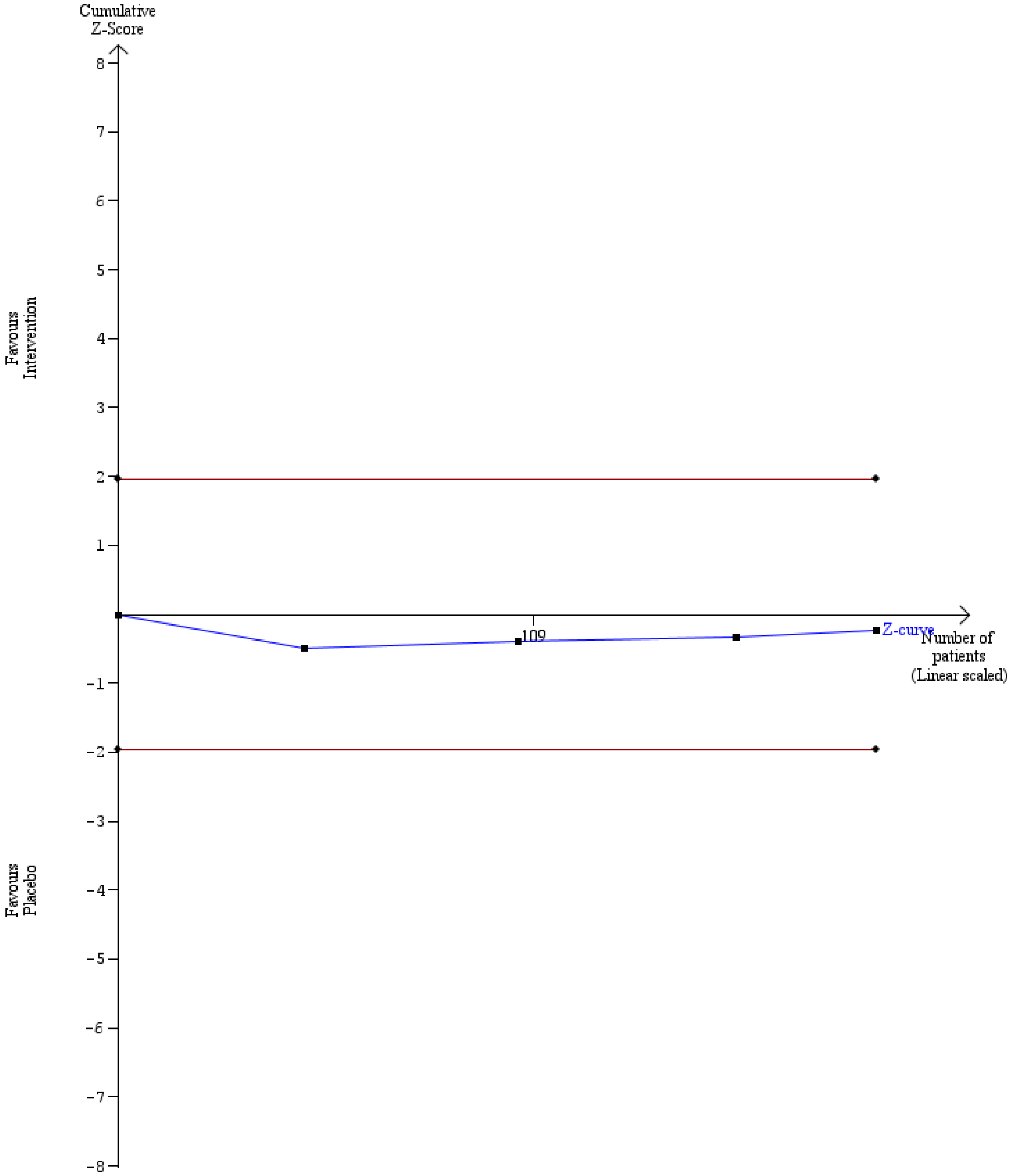

Supplementary Figure S3. Trial sequential analysis evaluating the robustness of the cumulative evidence for waist circumference.

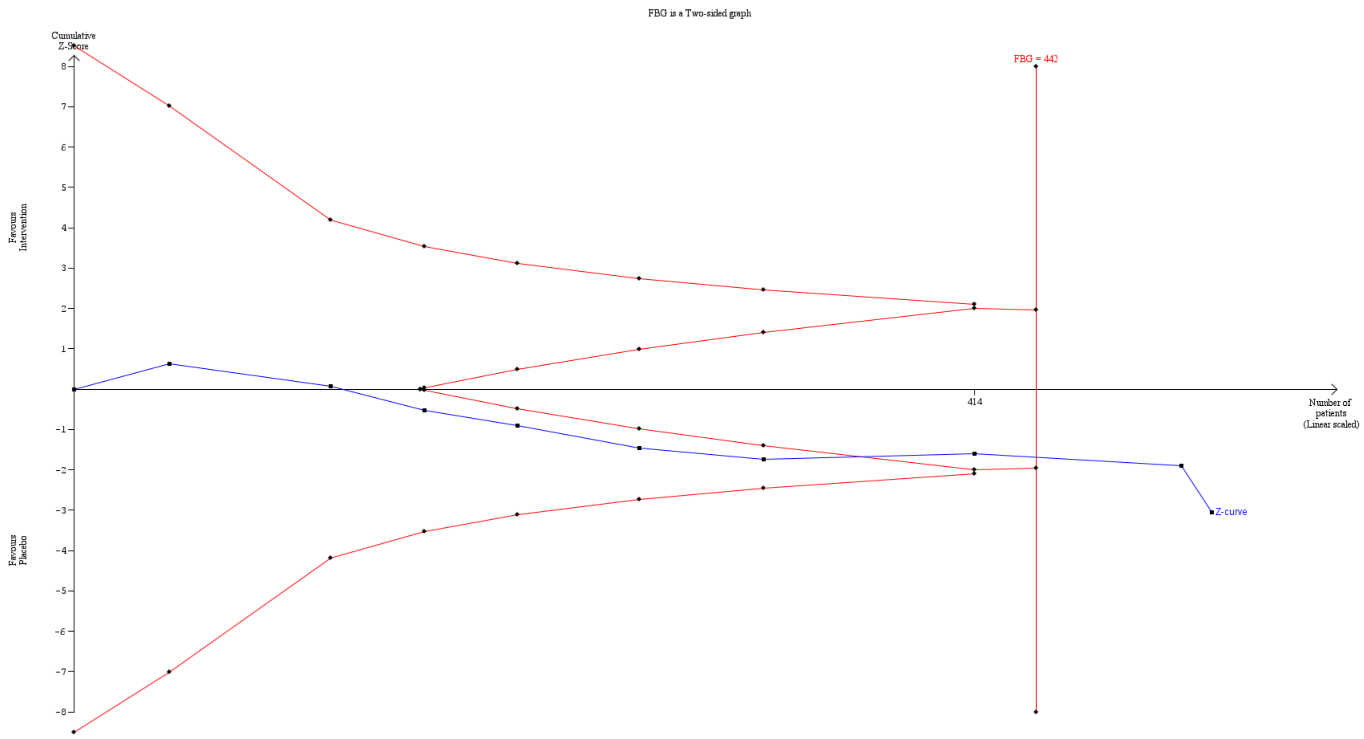

Supplementary Figure S4. Trial sequential analysis evaluating the robustness of the cumulative evidence for fasting blood glucose.

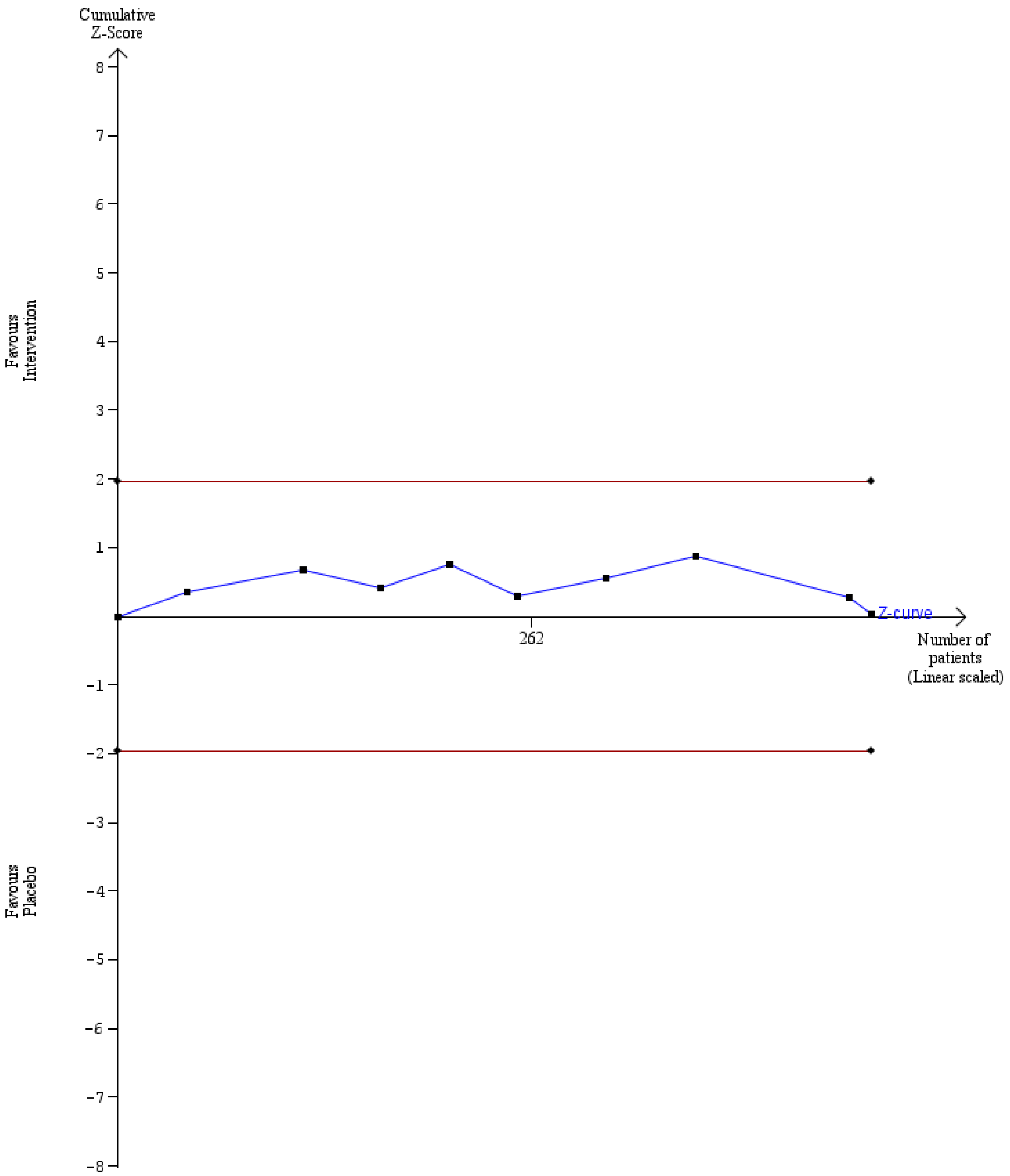

Supplementary Figure S5. Trial sequential analysis evaluating the robustness of the cumulative evidence for triacylglycerol levels.

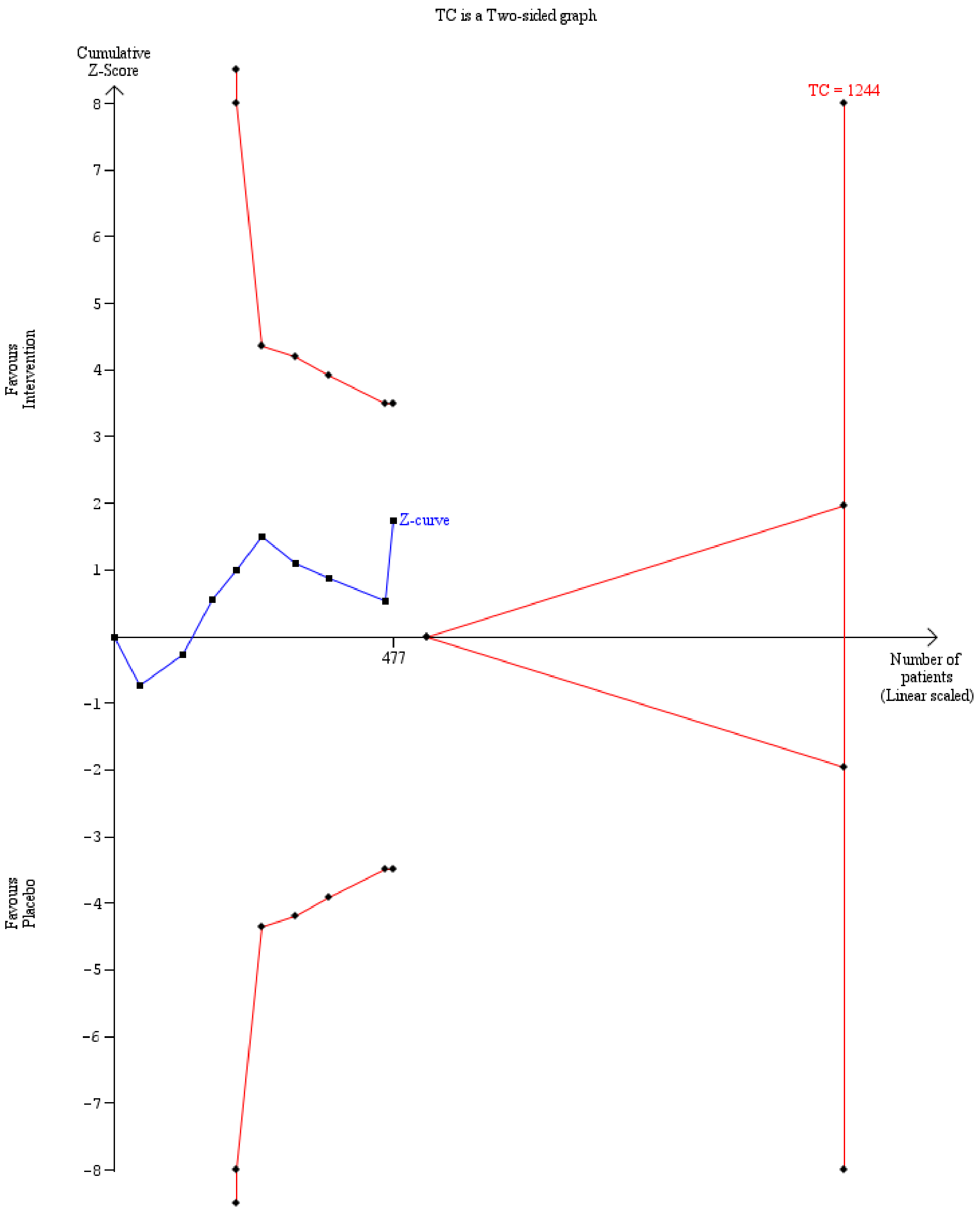

Supplementary Figure S6. Trial sequential analysis evaluating the robustness of the cumulative evidence for total cholesterol.

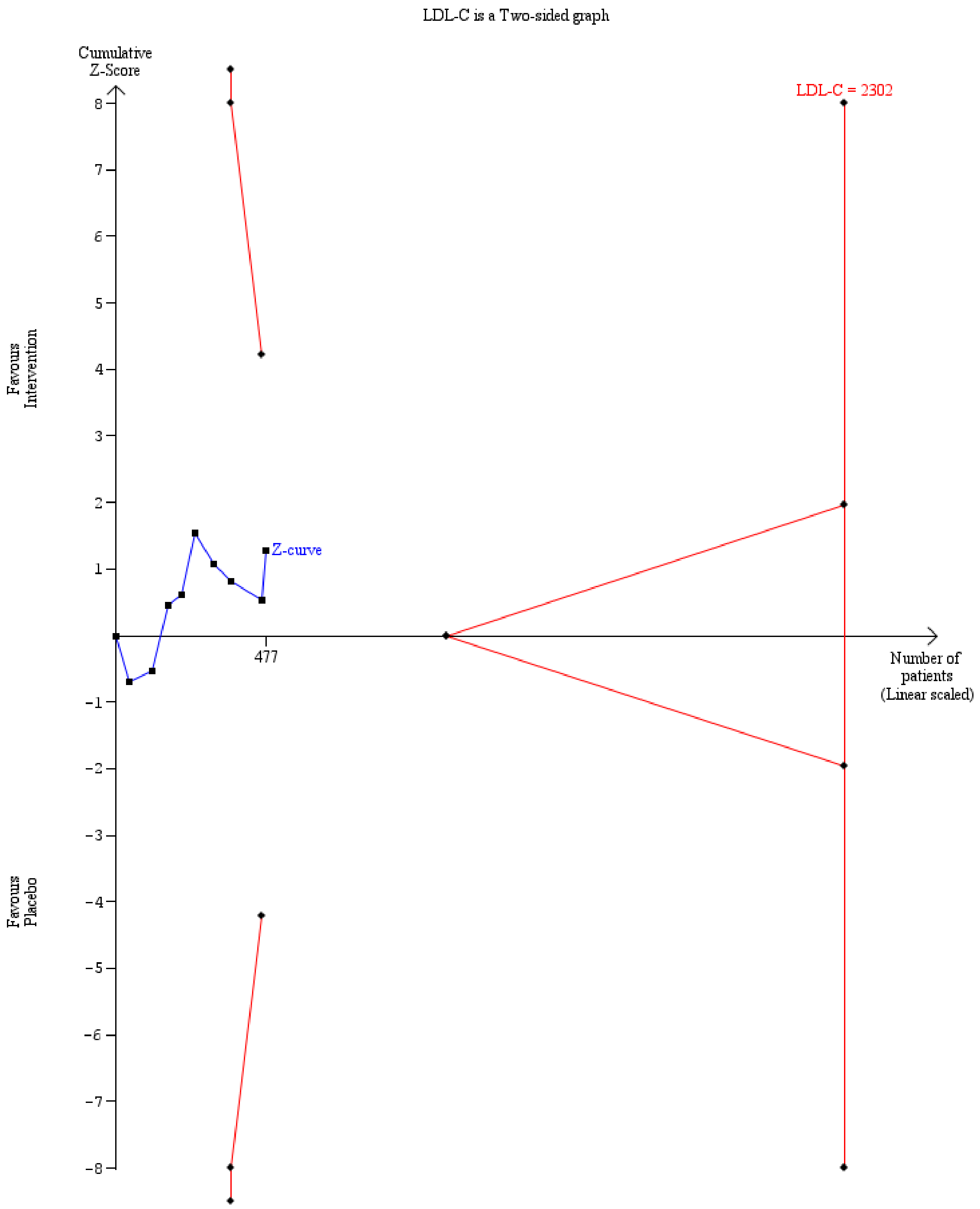

Supplementary Figure S7. Trial sequential analysis evaluating the robustness of the cumulative evidence for LDL-C.

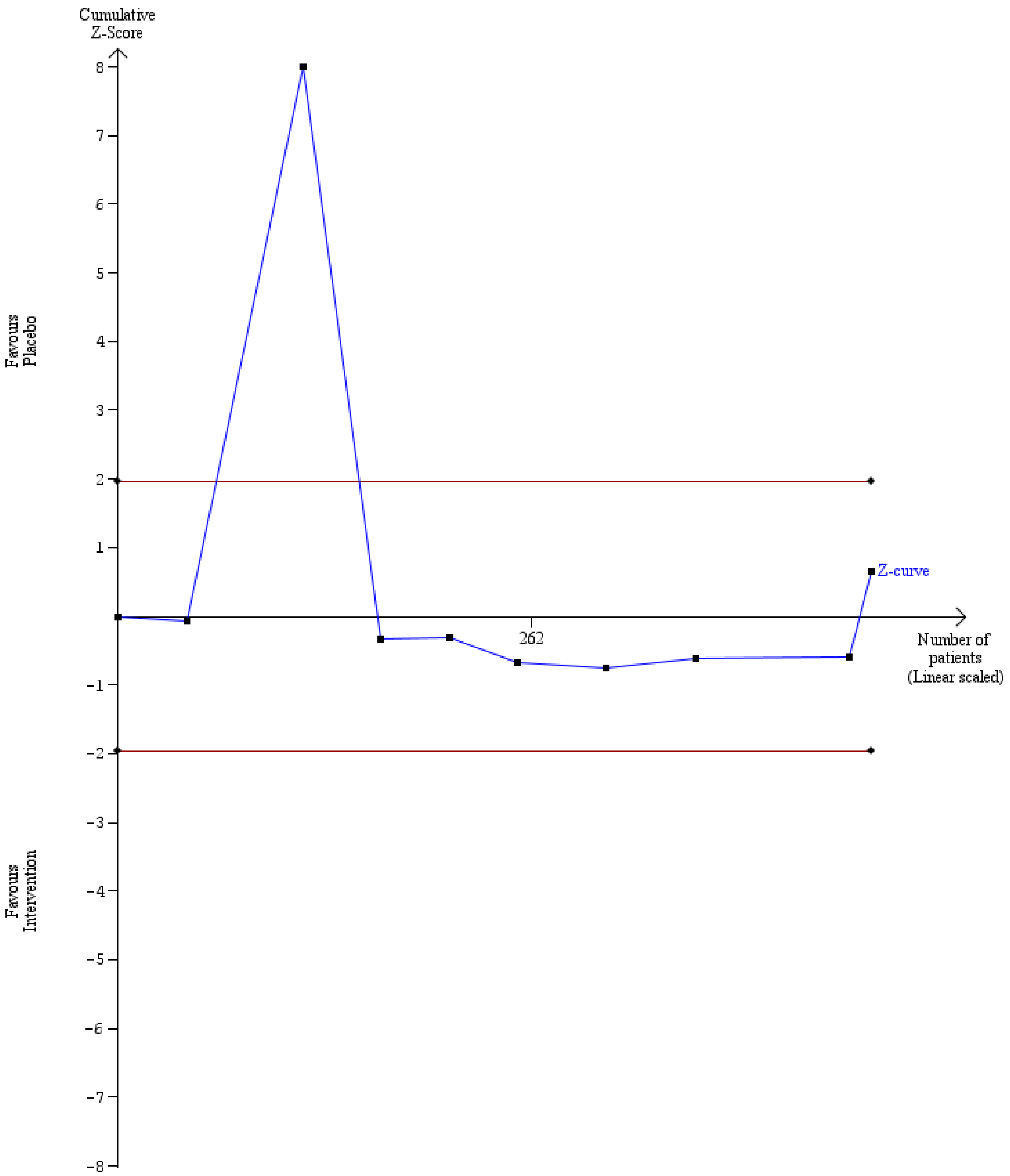

Supplementary Figure S8. Trial sequential analysis evaluating the robustness of the cumulative evidence for HDL-C.

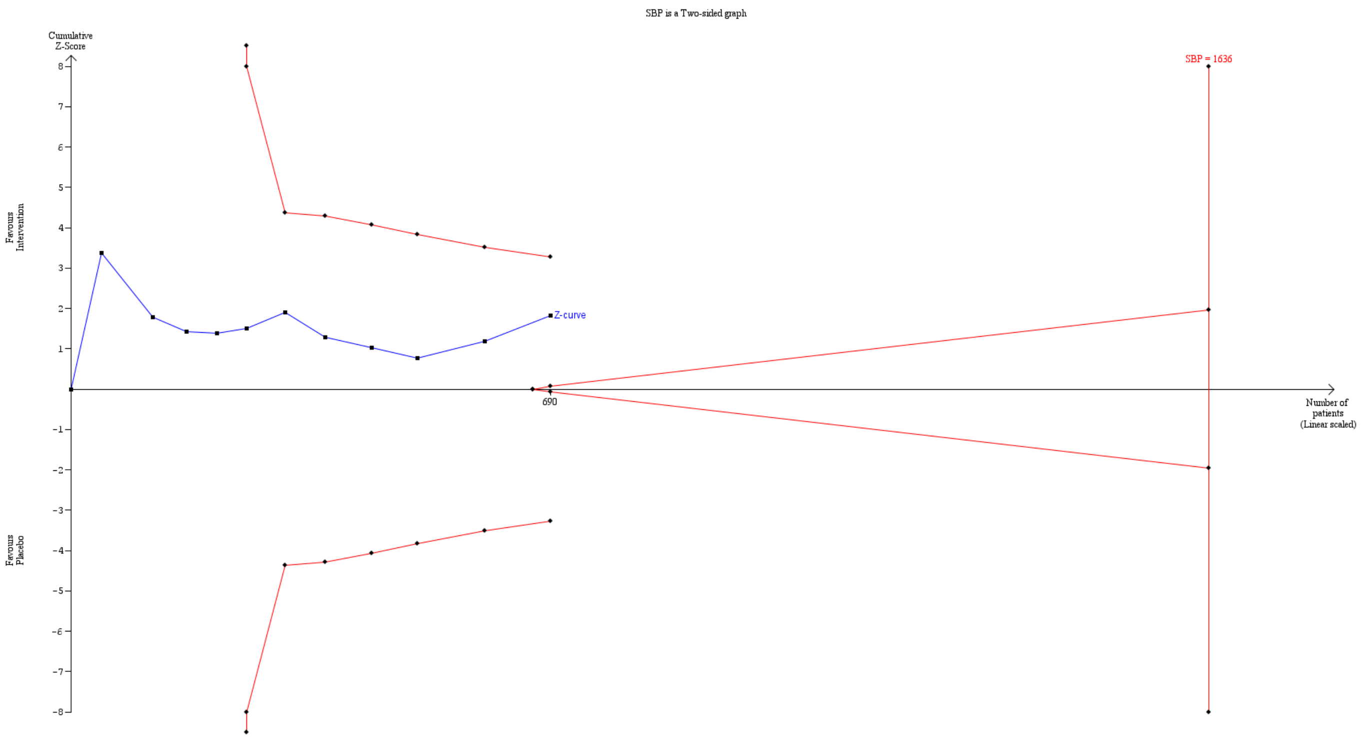

Supplementary Figure S9. Trial sequential analysis evaluating the robustness of the cumulative evidence for SBP.

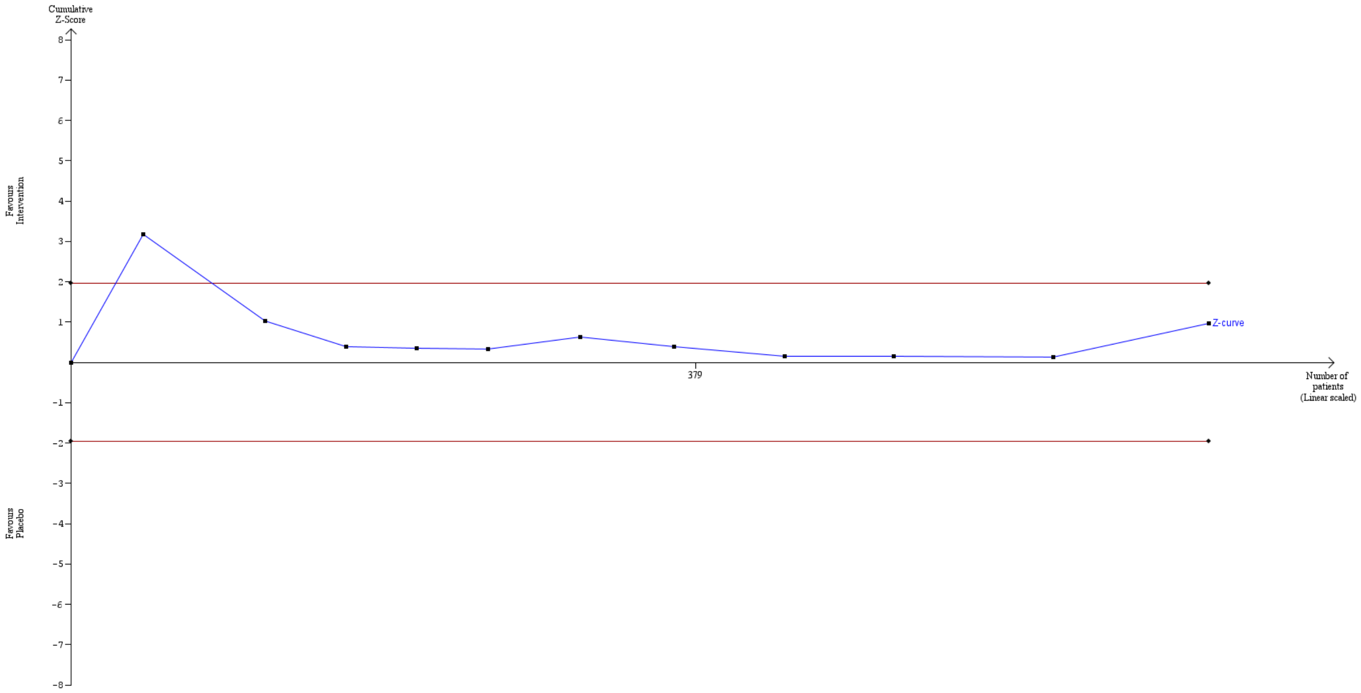

Supplementary Figure S10. Trial sequential analysis evaluating the robustness of the cumulative evidence for DBP.

Disclaimer/Publisher's Note: The statements, opinions and data contained in all publications are solely those of the individual author(s) and contributor(s) and not of MDPI and/or the editor(s). MDPI and/or the editor(s) disclaim responsibility for any injury to people or property resulting from any ideas, methods, instructions or products referred to in the content.
